# Xiao-Yin-Fang Therapy Alleviates Psoriasis-like Skin Inflammation Through Suppressing γδT17 Cell Polarization

**DOI:** 10.3389/fphar.2021.629513

**Published:** 2021-04-16

**Authors:** Xilin Zhang, Xiaorui Li, Youdong Chen, Bingjie Li, Chunyuan Guo, Peng Xu, Zengyang Yu, Yangfeng Ding, Yuling Shi, Jun Gu

**Affiliations:** ^1^ Department of Dermatology, Shanghai Skin Disease Hospital, School of Medicine, Tongji University, Shanghai, China; ^2^ Institute of Psoriasis, School of Medicine, Tongji University, Shanghai, China; ^3^ Department of Dermatology, Changhai Hospital, Second Military Medical University, Shanghai, China; ^4^ Department of Dermatology, Longhua Hospital, Shanghai University of Traditional Chinese Medicine, Shanghai, China; ^5^ Department of Dermatology, Shanghai Tenth People’s Hospital, School of Medicine, Tongji University, Shanghai, China

**Keywords:** psoriasis, traditional Chinese medicine, Xiao-Yin-Fang therapy, γδT cells, interleukin-17, relapse

## Abstract

Psoriasis is an immune-mediated chronic inflammatory skin disease primarily mediated by the activation of interleukin (IL)-17-producing T cells. Traditional Chinese Medicine (TCM) represents one of the most effective complementary and alternative medicine (CAM) agents for psoriasis, which provides treasured sources for the development of anti-psoriasis medications. Xiao-Yin-Fang (XYF) is an empirically developed TCM formula that has been used to treat psoriasis patients in Shanghai Changhai Hospital for over three decades. Imiquimod (IMQ)-induced psoriasis-like dermatitis mouse model was utilized to investigate the therapeutic effects of XYF by the assessment of disease severity and skin thickness. Flow cytometric assay was performed to explore the influence of XYF on skin-related immunocytes, primarily T cells. And, RNA sequencing analysis was employed to determine the alternation in gene expression upon XYF therapy. We discovered that XYF alleviated psoriasis-like skin inflammation mainly through suppressing dermal and draining lymph-node IL-17-producing γδT (γδT17) cell polarization. Moreover, XYF therapy ameliorated the relapse of psoriasis-like dermatitis and prohibited dermal γδT cell reactivation. Transcriptional analysis suggested that XYF might regulate various inflammatory signaling pathways and metabolic processes. In conclusion, our results clarified the therapeutic efficacy and inner mechanism of XYF therapy in psoriasis, which might promote its clinical application in psoriasis patients and facilitate the development of novel anti-psoriasis drugs based on the bioactive components of XYF.

## Introduction

Psoriasis is a common, recurrent inflammatory skin disease, which can be triggered in genetically susceptible individuals by diverse etiological factors, including infection, stress, trauma, medication, tobacco, and alcohol consumption (Schleicher, 2016). Men and women are evenly aﬀected at all ages with an estimated prevalence of psoriasis in adults and children ranging between 0.51 to 11.43% and 0–1.37%, respectively, (Michalek et al., 2017). Psoriasis and its associated comorbidities massively influence the physical and mental health of psoriasis patients, which bring about heavy socioeconomic burdens (Pilon et al., 2019).

Psoriasis is clinically characterized by erythematous plagues covered with lamellar silver scales, which pathological features involve abnormal keratinocyte proliferation and immune cell infiltration. The interplay between T lymphocytes, dendritic cells (DC) and keratinocytes, forming a self-perpetuating loop to amplify cutaneous inflammation, has been well described in psoriasis formation (Diani et al., 2015). During the process, the activation of interleukin (IL)-17-producing T cells, mainly including T helper 17 (Th17) cells and IL-17-producing γδT (γδT17) cells, play an essential role in the pathogenesis of psoriasis (Cai et al., 2013; Casciano et al., 2018).

The management of psoriasis comprises a variety of local and systemic therapies, involving phototherapy, retinoids, corticosteroids, vitamin D3 analogues, immunosuppressants, and recently emerged biologics targeting inflammatory factors, especially IL-17 (Kim and Krueger, 2017). Nevertheless, anti-psoriasis treatments are inevitably accompanied by adverse effects, such as irritation, skin atrophy, impaired hematopoiesis, visceral dysfunction and compromised immune function (Rapalli et al., 2018). A recent survey discovered that around half of the patients treated with oral therapy or biologics discontinued medication due to lack or loss of efficacy, tolerability reasons and safety issues, which signified that treatment dissatisfaction hindered the optimal care of psoriasis patients (Lebwohl et al., 2014). Hence, there is an urgent need to develop novel strategies and medicines for psoriasis management.

Complementary and alternative medicine (CAM) represents a group of various medical systems, practices, and products beyond conventional Western medication. Up to 62% of patients with psoriasis replaced or supplemented conventional therapies with CAM owing to their fewer side effects (Magin et al., 2006; Damevska et al., 2014; Murphy et al., 2019). The commonly utilized CAM for psoriasis includes Traditional Chinese Medicine (TCM), botanical therapy, vitamin supplement, dietary change, and behavioral intervention. Despite limited published evidence, TCM appeared to be the most effective CAM agent (Farahnik et al., 2017). TCM generally exploits topical or oral formulations, which are the mixtures of herbal, animal and/or mineral substances, and physical therapeutics, primarily including acupuncture and massage. Psoriasis was firstly recorded in the Treatise on the Causes and Manifestations of Diseases of Chinese Sui dynasty (581-618 CE) and has been efficaciously handled with TCM in China for over a thousand years. Therefore, TCM provides treasured sources for the development of anti-psoriasis medications.

Xiao-Yin-Fang (XYF), which is composed of *Isatis tinctoria L.*, *Scutellaria baicalensis Georgi*, *Salvia miltiorrhiza Bunge*, *Sophora flavescens Aiton*, and *Rheum officinale Baill.*, represents an empirically developed formula that originates from Doctor Ming Chen and Doctor Jun Gu. XYF has been prescribed to treat psoriasis patients for over three decades in Shanghai Changhai Hospital, which therapeutic efficacy has been proven by clinical research (Zhang et al., 2008; Wang et al., 2012b). Our previous research has shown that XYF decoction combined with calcipotriol ointment repressed peripheral T cell secretion of IL-17 in patients with psoriasis (Wang et al., 2012a). However, the pharmacological eﬀect and underlying mechanism of XYF in the treatment of psoriasis remained unclear.

In this study, we utilized imiquimod (IMQ)-induced psoriasis-like dermatitis mouse model to explore the therapeutic effect of XYF and its impact on the immune functions of conventional Th and γδT cells. We discovered that XYF alleviated psoriasis-like skin inflammation mainly through suppressing dermal and draining lymph-node (LN) γδT17 cell polarization. Moreover, XYF therapy ameliorated the relapse of psoriasis-like dermatitis and prohibited dermal γδT cell reactivation.

## Materials and Methods

### Mice

C57BL/6J mice were purchased from LINGCHANG Biotech (Shanghai, China), which were bred and maintained in specific pathogen-free (SPF) units with controlled temperature (22 ± 2°C), relative humidity (50 ± 5%), artificial light (12 h light/dark cycle) and free access to food/water in the animal facilities of Tongji University, Shanghai, China. Age-matched male mice (6–8 weeks of age; 18–22 g) were randomly used for all experiments in a non-blind manner. Handling of mice and experimental procedures were approved by the Animal Care and Use Committee of Shanghai Tongji University.

### Preparation of Xiao-Yin-Fang

Xiao-Yin-Fang (XYF) is an empirically developed Chinese medicine formula summarized and optimized by Chinese famous dermatologists Doctor Ming Chen and Doctor Jun Gu, which has been used to treat psoriasis in Shanghai Changhai Hospital. Its formula primarily includes five Chinese herbs: *Isatis tinctoria L.*, *Scutellaria baicalensis Georgi*, *Salvia miltiorrhiza Bunge*, *Sophora flavescens Aiton*, and *Rheum officinale Baill.* with a weight ratio of 30:15:30:10:3 (Table 1). The standardized powder of each herb, which was extracted under optimum condition and corresponded to a standard dosage of raw herb, was purchased from LongHua Hospital, University of Traditional Chinese Medicine, Shanghai, China. Voucher specimen of herb powder was deposited in Shanghai Skin Disease Hospital with reference No. XYF01-05, respectively. XYF powder was dissolved in double-distilled H_2_O, filtered through a 0.45 μm filter and stored at −20°C for subsequent application. All the procedures were in accordance with the rules and regulations of the 2015 Edition of China Pharmacopoeia.

**TABLE 1:** Constituents of Xiao-Yin-Fang (XYF).

Herb	Taxonomic nomenclature	Used parts	Weight (g)
Banlangen	*Isatis tinctoria L.*	Root	30
Huangqin	*Scutellaria baicalensis Georgi*	Root	15
Danshen	*Salvia miltiorrhiza Bunge*	Root	30
Kushen	*Sophora flavescens Aiton*	Root	10
Dahuang	*Rheum officinale Baill.*	Root	3

### Qualitative and Quantitative Analysis of Xiao-Yin-Fang by UPLC-Q/TOF-MS

Chemical profiling of XYF was performed on an Agilent 1290 UPLC System (Agilent Technologies Co., Santa Clara, CA, United States) coupled with AB SCIEX Triple TOF 4600^®^ quadrupole time-of-flight mass spectrometry (AB SCIEX, Foster City, CA, United States). Chromatographic separation was conducted on an Acquity UPLC^®^ HSS T3 Column (2.1 × 100 mm i.d., 1.8 μm; Waters, Milford, MA, United States) at 30°C, using a mixed mobile phase consisting of 0.1 formic acid in water (mobile phase A) and 0.1 formic acid in acetonitrile (mobile phase B). The gradient elution program was used as follows: 0–5 min, 0–0% B; 5–7 min, 0–3% B; 7–15 min, 3–5% B; 15–17 min, 5–16% B; 17–19 min, 16–17% B; 19–30 min, 17% B; 30–34 min, 17–20% B; 34–42 min, 20% B; 42–44 min, 20–22% B; 44–49 min, 22% B; 49–54 min, 22–60% B; 54–58 min; 60–95% B; 58–60 min, 95% B; 60–60.1 min, 95–0% B; 60.1–63 min, 0% B. The flow rate was 0.3 ml/min, and the sample injection volume was 1 μl. Data acquisition was performed on AB SCIEX Triple TOF 4600^®^ system equipped with an electrospray ionization (ESI) source in the negative and positive ion modes. The mass spectrometry was operated in full-scan TOF-MS (m/z 50-1700) and information-dependent acquisition (IDA) MS/MS modes. The parameters of mass spectrometry were as follows: ion source gas 1 and 2 were 50 psi; curtain gas was 35 psi; ion source temperature was 500°C; ion spray voltage floating was 5000 V (positive)/4500 V (negative). In the tandem mass spectrometry (MS/MS) experiments, mass range was 50–1250, collision energy was 40 ± 20 eV, ion release delay was 30 ms and ion release width was 15 ms. All data acquisition and processing were validated using Analyst TF 1.7.1 software (AB SCIEX).

For content determination, seven standards of representative components from five constitutive herbs of XYF, including (R, S)-goitrin (#8508), baicalin (#5719), salvianolic acid B (#4065), tanshinone II a (#7503), matrine (#844), oxymatrine (#8678), and emodin (#8171), were purchased from Standard Technology Co., Shanghai, China (http://www.nature-standard.com/). Quantitative analysis of these components in XYF by UPLC-DAD was performed on a Waters H-Class UPLC system (Waters, Milford, MA, United States) as previously reported (Xiang et al., 2018). UV detector was monitored at 225 nm (matrine/oxymatrine), 245 nm ((R, S)-goitrin), 254 nm (emodin), 270 nm (tanshinone II a), 280 nm (baicalin) and 286 nm (salvianolic acid B).

### Imiquimod-Induced Psoriasis-like Skin Inflammation

Mice received a daily topical dose of 50 mg of imiquimod (IMQ) cream (5%; #H20030128, Sichuan Med-Shine Pharmaceutical) or control Vaseline (VAS; #180102, Shandong Mint) on shaved back (an approximate size of 2 × 3 cm^2^) or bilateral ears for five consecutive days. For the induction of disease relapse, 25 mg of IMQ was applied on mouse left ear once daily from day 0 to day 4, and 25 mg of IMQ was reapplied on mouse right ear once daily from day 12 to day 16. Mouse skin inflammation was evaluated by cumulative psoriasis area and severity index (PASI) score, calculated by the adding up of erythema score (0–4), infiltration score (0–4), and desquamation score (0–4). The severity of each symptom was assessed comparing with reference pictures. Skin thickness was recorded as the average value of three measurements by vernier calipers at the center of mouse dorsal or ear lesion before topical treatment.

### Experiment Design and Drug Administration

Mice were randomly allocated into the following groups: control group, model group, low-dose XYF group, medium-dose XYF group, high-dose XYF group, and multi-glycoside of Tripterygium wilfordii Hook. f (GTW) group. GTW tablets (#Z42021212, Hubei Huangshi Yunfei Pharmaceutical Company) were ground down and dissolved in ddH_2_O, which were filtered and stored at −20 °C. Based on drug-dose conversion between human and mouse, 200 μl of XYF decoction (low-dose: 5.8 mg/g; medium-dose: 11.6 mg/g; high-dose: 23.3 mg/g), GTW (7.89 μg/g) or ddH_2_O was administered once daily by gavage for 10 successive days, and VAS or IMQ was topically applied from day 6 to day 10.

### Immunohistochemical Analysis

Paraffin-embedded skin specimens were prepared by routine methods, and the sections were stained by hematoxylin-eosin (H&E) with additional immunostaining for Ki-67. The sections were deparaffinized with xylene and rehydrated through the incubation with graded alcohol into water. For H&E staining, the sections were then stained with hematoxylin, washed with PBS, differentiated with hydrochloric acid ethanol, and stained with eosin. Pathological change was examined under the Olympus CX33/BX53 optical microscope (Olympus, Southborough, MA, United States). Epidermal hyperplasia (acanthosis) was measured as the average length between the basement membrane and the stratum corneum. Papillomatosis index is the ratio of the length of the dermal-epidermal junction to the surface length of the epidermis. For Ki-67 staining, heat-induced epitope retrieval was performed in EDTA buffer (#RC016, RecordBio) at pH 8.3% hydrogen peroxide was utilized to block endogenous peroxidase activity. The sections were then incubated with anti-Ki-67 antibody (#ab16667, Abcam) overnight at 4°C, followed by incubation with REAL EnVision and visualized by DAB^+^ (#K5007, DAKO). Ki-67 staining was evaluated semi-quantitatively according to the expression level of cytoplasmic brown staining in five random epidermal fields under 400x magnification performed independently by two researchers. The intensity of staining (IS, intensity score) was assessed as: absent (0), weak (1), moderate (2), and strong (3). The percentage of stained cells (PS, proportion score) was scored: 0–5% (0), 6–25% (1), 26–50% (2), 51–75% (3), and 75–100% (4). H-score was computed as the average value of the multiplication of IS and PS and interpreted by the following way: 0 as negative staining, 1 to 4 as weak positive staining, 5 to 8 as medium positive staining and 9 to 12 as strong positive staining.

### Enzyme-Linked Immunosorbent Assay

IL-17 levels in the serum samples collected from experimental mice were measured using mouse IL-17A ELISA kit (RayBio, Norcross, GA, United States) according to the manufacturers’ instructions.

### Dermal Single-Cell Suspension Preparation

Mouse ears were harvested and incubated in dispase II (5 mg/ml; #D4693, Sigma) for 1 h at 37°C. Dermal sheet was peeled from epidermal sheet, cut into little pieces, and digested at 37°C in DMEM (#SH30022.01, Hyclone) containing collagenase IV (1 mg/ml; #SH30256.01B, Hyclone) and DNase (0.015 mg/ml; #B002004, Diamond) for one and a half hour. Dermal single-cell suspension was harvested by passing through a 40 μm cell strainer (#CSS-010-040, Biofil).

### *In Vitro* PMA and Ionomycin Activation Assay

T cell culture medium was RPMI 1640 (#11875119, Gibco) supplemented with heat-inactivated fetal bovine serum (10%; #SH30084.03, Hyclone), penicillin G (100U/ml; #B540733, Sangon), streptomycin sulfate (100 μg/ml; #B540733, Sangon) and amphotericin B (2.5 μg/ml; #B540733, Sangon). Mouse skin-draining axillary, brachial and inguinal lymph nodes (LN) were harvested. LN single-cell suspensions were prepared through grinding by glass slides and filtering through a 40 μm cell strainer. LN and dermal cells were cultured in T cell culture medium in the presence of phorbol-12-myristate-13-acetate (PMA; 50 ng/ml; #P1585, Sigma), ionomycin (1mM; #abs42019871, Absin) and brefeldin A (1:1000; #347688, BD) for 4 h.

### Flow Cytometric Analysis

Single-cell suspensions were preincubated with anti-CD16/CD32 antibodies (Ab; clone 2.4G2; #553141, BD Biosciences) for 15 min at 4°C. To discriminate viable cells from dead cells, cells were stained with fixable viability stain (#564406, BD Biosciences) for 10 min at room temperature. For the analysis of surface markers, cells were stained with anti-CD3 Ab (clone 145-2C1; #553061, BD Biosciences), anti-CD4 Ab (clone GK1.5; #562891, BD Biosciences), anti-CD25 Ab (clone 3C7; #101904, Biolegend), anti-CD45 Ab (clone 30-F11; #103134, Biolegend), anti-CD11b Ab (clone M1/70; #553310/#564455, BD Biosciences), anti-TCRβ Ab (clone H57–597; #560657, BD Biosciences), anti-γδTCR Ab (clone GL3; #118115, Biolegend), anti-MHC-II Ab (clone M5/114.15.2; #563414, BD Biosciences), anti-F4/80 Ab (clone T45-2342; #565410, BD Biosciences), anti-CD11c Ab (clone HL3; #566505, BD Biosciences), anti-Ly6C Ab (clone AL-21; #553104, BD Biosciences), and anti-Ly6G Ab (clone 1A8; #560599, BD Biosciences) for 30 min at 4°C protected from light. For cytoplasmic staining, cells were fixed and permeabilized with BD Cytofix/Cytoperm (#51-2090KZ, BD Biosciences) and were stained with anti-mouse IFN-γ Ab (clone XMG1.2; #505810, Biolegend), anti-mouse IL-4 Ab (clone 11B11; #560699, BD Biosciences), anti-mouse IL-17 Ab (clone TC11-18H10; #559502, BD Biosciences) and incubated for 30 min at 4°C. For intranuclear staining, cells were fixed and permeabilized with Foxp3/Transcription Factor Fixation/Permeabilization Concentrate and Diluent (#00-5521-00, eBioscience), and were then stained with anti-mouse Foxp3 Ab (clone MF-14; #126408, Biolegend), anti-Ki-67 Ab (clone B56; #556027, BD Biosciences) and anti-ROR-γt Ab (clone Q31-378; #564723, BD Biosciences). For Annexin-V staining, cells were resuspended in 1x annexin-binding buffer (#556454, BD Biosciences) and stained with Annexin-V (#640908, Biolegend) for 15 min at room temperature. Finally, cells were assayed with BD LSRFortessa Cytometer, and analyzed with FlowJo software (Treestar). Gate strategy of LN CD4^+^ T cells, LN γδT cells, dermal neutrophils, dermal inflammatory monocytes, dermal CD4^+^ T cells, and dermal γδT cells were demonstrated in Supplementary Figure S3.

### RNA Sequencing Analysis

Total RNA samples were prepared from intact epidermal or dermal ear sheets of mouse ear skin from two mice. Double-strand cDNA was generated from equal amounts of total RNA by following TruSeq8 RNA Library Prep Kit v2 (#RS-122-2001/2002, Illumina). The cDNA libraries were sequenced using Illumina Hi-seq2500. STAR software was utilized for sequence alignment between the preprocessing sequence and reference genome sequence of mice downloaded from the Ensembl database (Mus_musculus.GRCm38.90,ftp://ftp.ensembl.org/pub/release-90/gtf/mus_musculus/Mus_musculus.GRCm38.90. chr.gtf.gz). Transcript assembly of mRNA sequencing data was performed by StringTie software. DESeq 2 was applied to conduct the analysis of differentially expressed genes (DEG). The cutoffs of DEG were determined as the adjusted *P* value ≤0.05 and the |log_2_FC| ≥ 1. Functional annotations of the DEGs were conducted using Kyoto Encyclopedia of Genes and Genomes (KEGG) pathways analysis and Gene Set Enrichment Analysis (GSEA).

### Statistical Analysis

GraphPad Prism 6.0 software was utilized for statistical analysis. Student's t-test or one-way ANOVA were utilized to analyze the differences between the groups following Gaussian distributions with homogeneity of variance. Data that did not follow Gaussian distributions were analyzed using the Kruskal-Wallis test. Differences were statistically significant when *P* < 0.05.

## Results

### UPLC-Q/TOF-MS Analysis of Xiao-Yin-Fang

**FIGURE 1:**
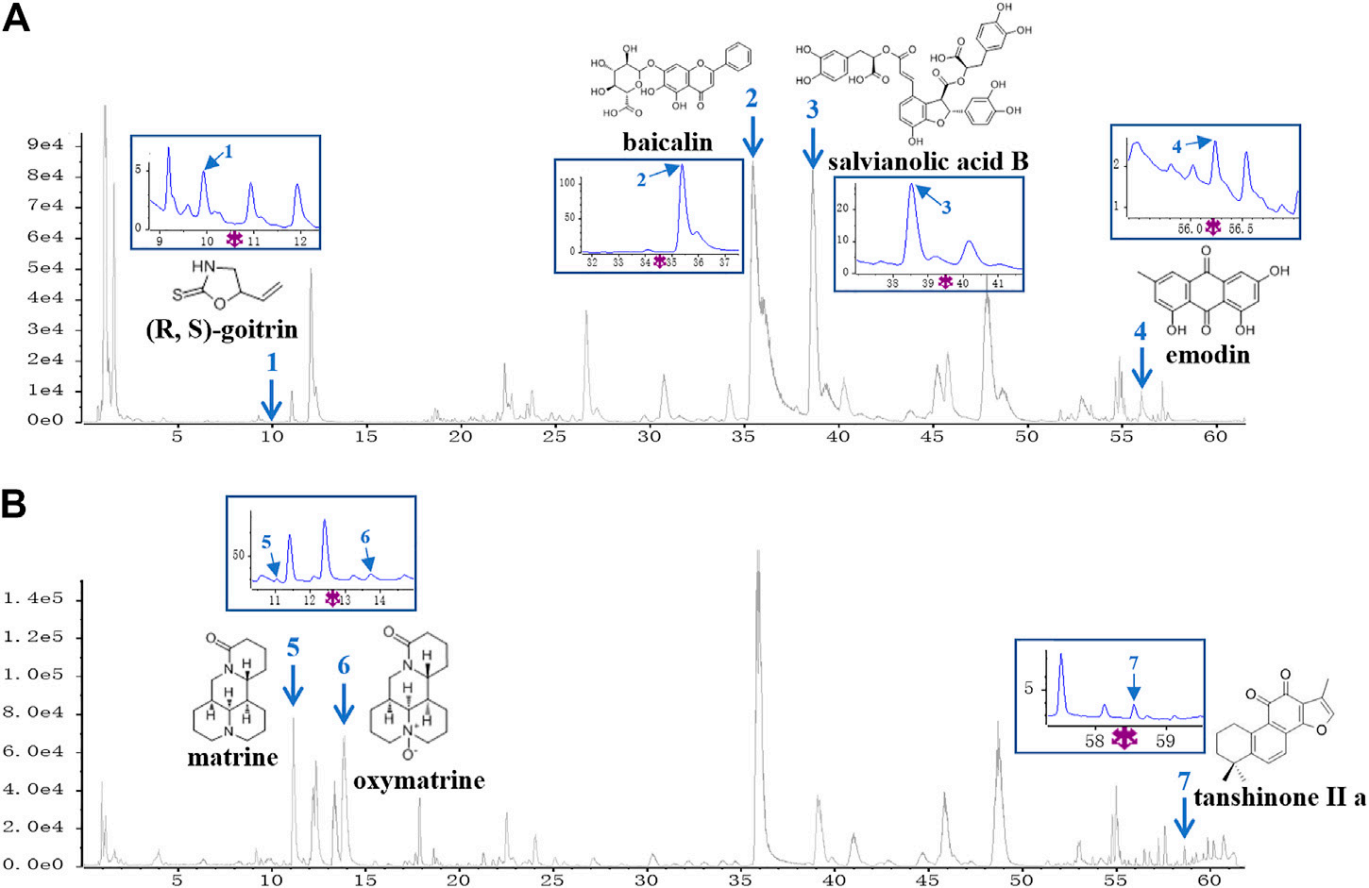
UPLC-Q/TOF-MS analysis of Xiao-Yin-Fang. Base Peak Chromatograms of Xiao-Yin-Fang (XYF) in ESI-negative mode **(A)** and ESI-positive mode **(B)** together with the chemical structures and ultraviolet (UV) chromatograms of seven components (1, (R, S)-goitrin; 2, baicalin; 3, salvianolic acid B; 4, emodin; 5, matrine; 6, oxymatrine; 7, tanshinone II a).

A UPLC-Q/TOF-MS method in both negative and positive ion modes was employed to rapidly characterize the major constituents in XYF. A total of 57 compounds were unambiguously or tentatively characterized by comparing their retention times and MS data with reference standards or with data reported in the literature (Supplementary Figure S1). These compounds were all derived from five medicinal materials that composed XYF, and did not contain any conventional immunosuppressants. Seven representative chemical compounds were chosen as chemical markers and quantified to evaluate the quality of relevant medicinal materials, including (R, S)-goitrin (1, Rt 9.928 min), baicalin (2, Rt 35.44 min), salvianolic acid B (3, Rt 38.63 min), tanshinone II a (7, Rt 58.63 min), matrine (5, Rt 11.03 min), oxymatrine (6, Rt 13.75 min), and emodin (4, Rt 56.12 min) (Figure 1). Quantitative determination of these seven compounds by UPLC-DAD was performed on a Waters H-Class UPLC system at different wavelengths. As a result, the contents of these seven compounds in XYF were 0.132 mg/g, 12.626 mg/g, 6.154 mg/g, 0.014 mg/g, 2.514 mg/g, 1.757 mg/g, and 0.007 mg/g, respectively.

### Xiao-Yin-Fang Alleviates Imiquimod-Induced Psoriasis-like Skin Inflammation

**FIGURE 2:**
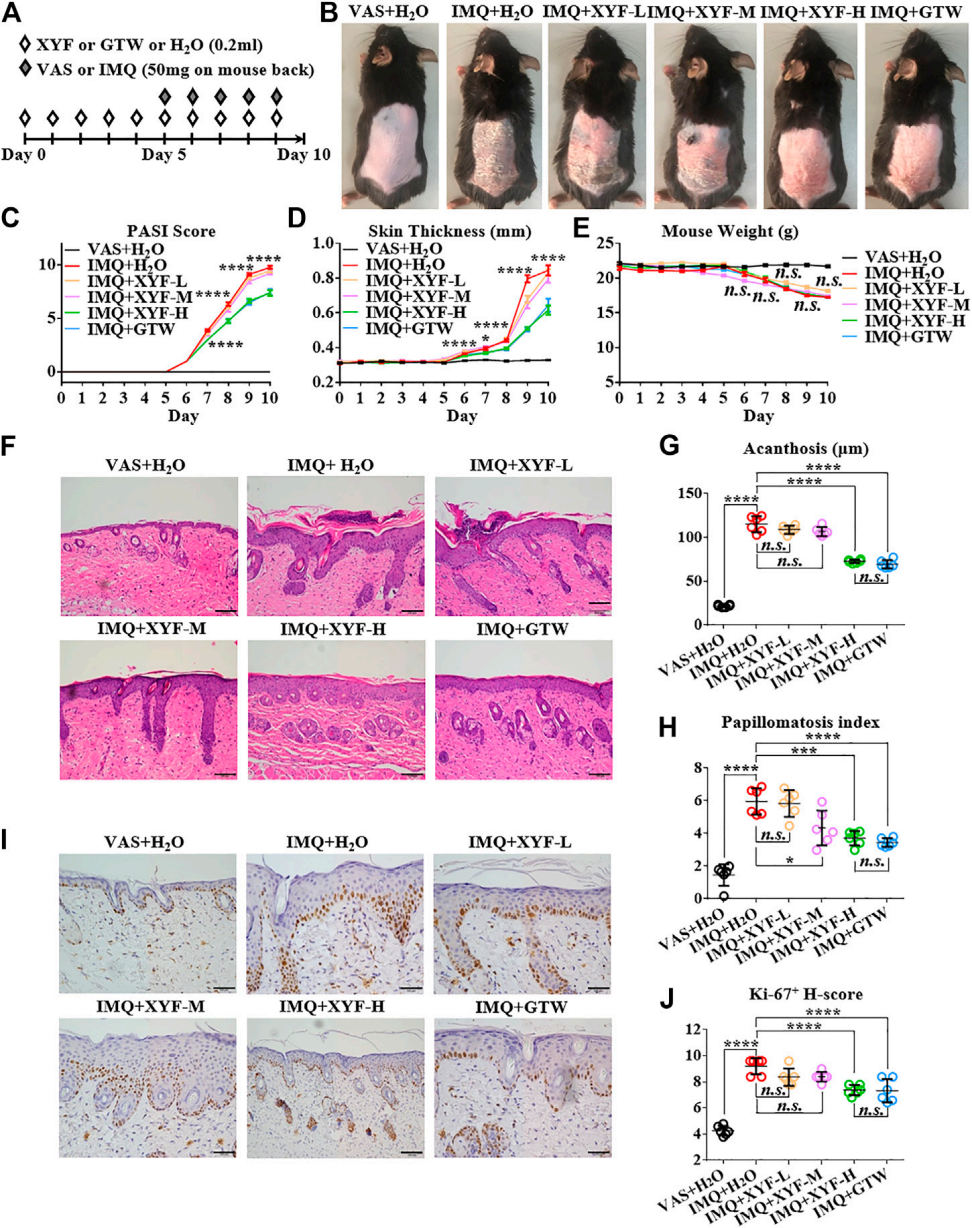
High-dose Xiao-Yin-Fang alleviated imiquimod-induced psoriasis-like skin inflammation on mouse back. **(A)** Schematic of the experimental procedures. Mice were given different-dose Xiao-Yin-Fang (XYF), multi-glycoside of *Tripterygium wilfordii Hook. f.* (GTW) solution or distilled water (H_2_O) by gavage twice daily from day 0 to day 9, and received imiquimod (IMQ) or Vaseline (VAS) application on shaved back skin once a day from day 5 to day 9. **(B)** Representative pictures of mouse skin lesions (XYF-L, low-dose XYL; XYF-M, medium-dose XYF; XYF-H, high-dose XYF). **(C–E)** Evaluation of PASI score **(C)**, skin thickness **(D)** and body weight **(E)** of mice. The statistic differences between IMQ + XYF-H group and IMQ + H_2_O group were annotated (*n* = 54, three independent experiments). **(F)** H&E staining and calculated epidermal acanthosis **(G)** and papillomatosis index **(H)** (x200; bar = 100 μm; *n* = 36, three independent experiments). **(I)** Ki-67 staining and assessment of its H-score of epidermal fields **(J)** (x200; bar = 100 μm; *n* = 36, three independent experiments). The data were presented as mean ± s.e.m.

To explore the potential mechanism of XYF in psoriasis, we utilized IMQ-induced psoriasis-like dermatitis mouse model. Mice were divided into six groups and treated as depicted in Figure 2A. Multi-glycoside of *Tripterygium wilfordii Hook. f* (GTW), also termed as Tripterygium glycosides, is a widely acknowledged efficacious psoriasis-treating TCM agent and has been proven to ameliorate murine psoriasis-like dermatitis, which was therefore chosen as positive drug in our study (Han et al., 2012; Wu et al., 2015a; Zhao et al., 2016; Lv et al., 2018; Nguyen et al., 2020a; Ru et al., 2020). As modeling with IMQ generated skin lesions resembling human plaque psoriasis, mice pretreated with XYF displayed lighter erythema, smoother skin, and thinner scales than mice treated with IMQ alone (Figure 2B). Moreover, XYF exerted therapeutic effects in a dose-dependent manner that high-dose XYF was as potent as GTW in alleviating psoriasis-like dermatitis (Figure 2B). These findings were verified by the evaluation of PASI score (Figure 2C) and back skin thickness (Figure 2D). Besides, the application of XYF did not cause any behavioral abnormality or extra weight loss in mice (Figure 2E). Subcutaneous vessel dilation was also less prominent in the mice from high-dose XYF group than model group (Supplementary Figure S2). In accordance, the histological examination showed that both high-dose XYF and GTW significantly reduced epidermal acanthosis and elevated papillomatosis index in an equal manner (Figures 2F–H). As the expression of Ki-67 in keratinocytes was upregulated in model mice, high-dose XYF group considerably decreased Ki-67^+^ H-score to a similar degree as GTW (Figures 2I,J). Overall, XYF therapy substantially alleviated IMQ-induced psoriasis-like skin inflammation when administered at the high dosage, which demonstrated a comparable efficacy as GTW. Therefore, high-dose XYF was employed in the following study.

### Xiao-Yin-Fang Prevents Lymphnode γδT Cell Secretion of IL-17

**FIGURE 3:**
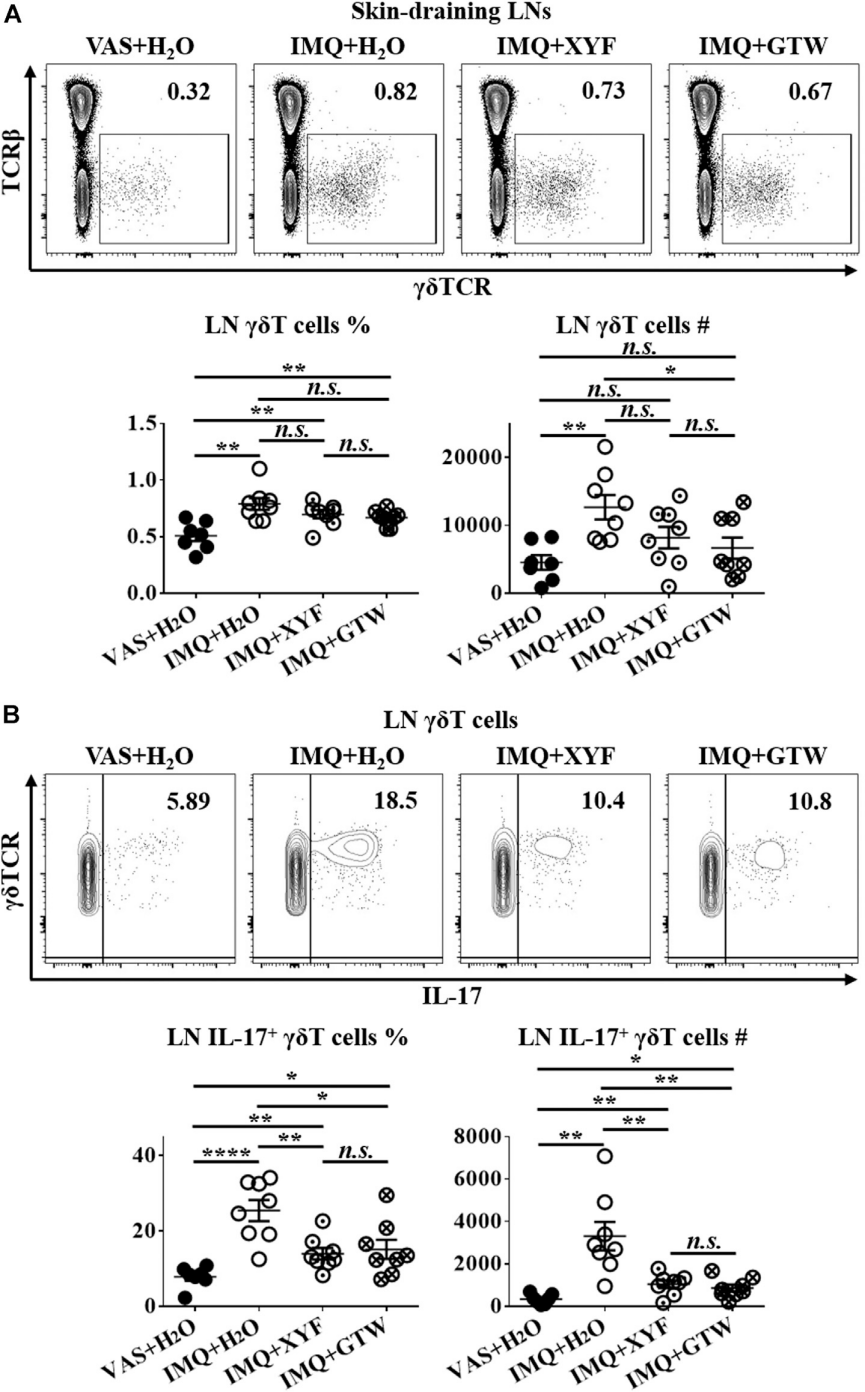
Xiao-Yin-Fang therapy prevented lymphnode γδT cell secretion of IL-17. Mice were treated as in Figure 2. Skin-draining axillary, brachial and inguinal lymph nodes (LN) were harvested on day 10. Freshly isolated LN cells were *in vitro* cultured in the presence of PMA, ionomycin and Brefeldin A for 4 h. LN cells were then stained with anti-CD45, TCRβ, γδTCR, CD11b, and IL-17 antibodies, which were analyzed by flow cytometry. Representative scatter plots, the ratios, and numbers of LN γδT cells **(A)** and IL-17^+^ γδT cells **(B)**. *n* = 31, three independent experiments. The data were presented as mean ± s.e.m.

Since T lymphocytes play a vital role in the pathogenesis of psoriasis, we next sought to investigate the impact of XYF on T cell subsets within skin-draining lymph nodes (LN). Compared with control mice, we detected a decreased ratio of LN CD4^+^ T cells with greater IL-17 production in model mice (Supplementary Figure S4A–D). The proportion of regulatory T (Treg; CD25^+^Foxp3^+^) cells in CD4^+^ T cells was also increased (Supplementary Figure S4E). However, neither XYF nor GTW disturb the balance in Th and Treg cells (Supplementary Figure S4). While serum IL-17 levels were upregulated in model mice, the application of XYF reduced the serum contents of IL-17 (Supplementary Figure S5). Previous research demonstrated that γδT cells are the main source of IL-17 in psoriasis-like dermatitis (Cai et al., 2011). Accordantly, the percentage and number of LN γδT cells were substantially elevated in model mice (Figure 3A), and their secretion of IL-17 was raised approximately threefold (Figure 3B). Although XYF failed to suppress the expansion of LN γδT cells (Figure 3A), it drastically hindered their polarization into γδT17 cells to a comparable extent as GTW (Figure 3B). Hence, XYF therapy might exert its anti-inflammatory role in psoriasis-like dermatitis mainly through its inhibition of γδT17 polarization.

### Xiao-Yin-Fang Suppresses Dermal γδT17 Cell Polarization

**FIGURE 4:**
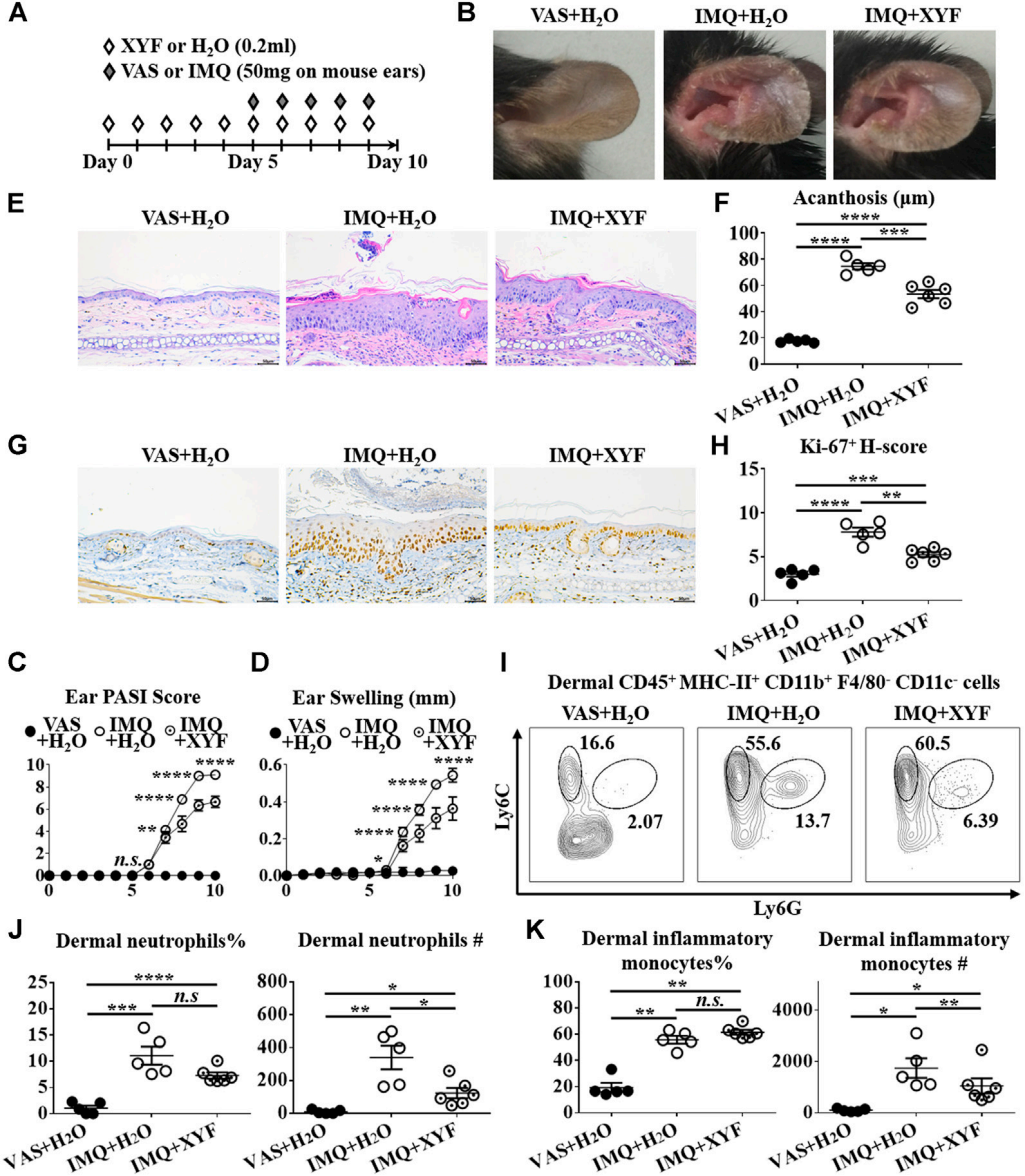
Xiao-Yin-Fang lessened imiquimod-induced psoriasis-like dermatitis on mouse ears. **(A)** Schematic of the experimental procedures. Mice were given XYF or H_2_O by gavage twice daily from day 0 to day 9, and received IMQ or VAS application on mouse ears once a day from day 5 to day 9 **(B)** Representative pictures of mouse ear lesions. **(C, D)** Evaluation of PASI score **(C)** and skin swelling **(D)** of mouse ears (*n* = 27, three independent experiments) **(E)** H&E staining and calculated epidermal acanthosis **(F)** (x200; bar = 50 μm; *n* = 18, three independent experiments). **(G)** Ki-67 staining and assessment of its H-score of epidermal fields **(H)** (x200; bar = 50 μm; *n* = 18, three independent experiments). **(I–K)** Freshly isolated dermal cells were obtained and stained with anti-CD45, MHC-II, CD11b, F4/80, CD11c, Ly6C, and Ly6G antibodies, which were analyzed by flow cytometry. **(I)** Representative scatter plots of dermal neutrophils and inflammatory monocytes. The ratios and cell numbers of dermal neutrophils **(J)** and inflammatory monocytes **(K)** (*n* = 16, three independent experiments). The data were presented as mean ± s.e.m.

Given that skin-resident γδT cells elicited direct action in local inflammation, we established the model of psoriasis-like dermatitis on mouse ears to assess the role of dermal γδT cells in the curative effects of XYF (Figure 4A). As depicted in Figures 4B–H, XYF significantly reduced the disease severity of psoriasis-like dermatitis on mouse ears. While neutrophils (Ly6C^+^Ly6G^+^) and inflammatory monocytes (Ly6C^+^Ly6G^−^) gathered in the dermis following IMQ application, XYF considerably lowered their cell numbers rather than their percentages (Figures 4I–K), corroborating the anti-psoriasis effect of XYF. In line with the findings of LNs, XYF did not affect the homeostasis and function of dermal conventional T cells (Supplementary Figure S6).

**FIGURE 5:**
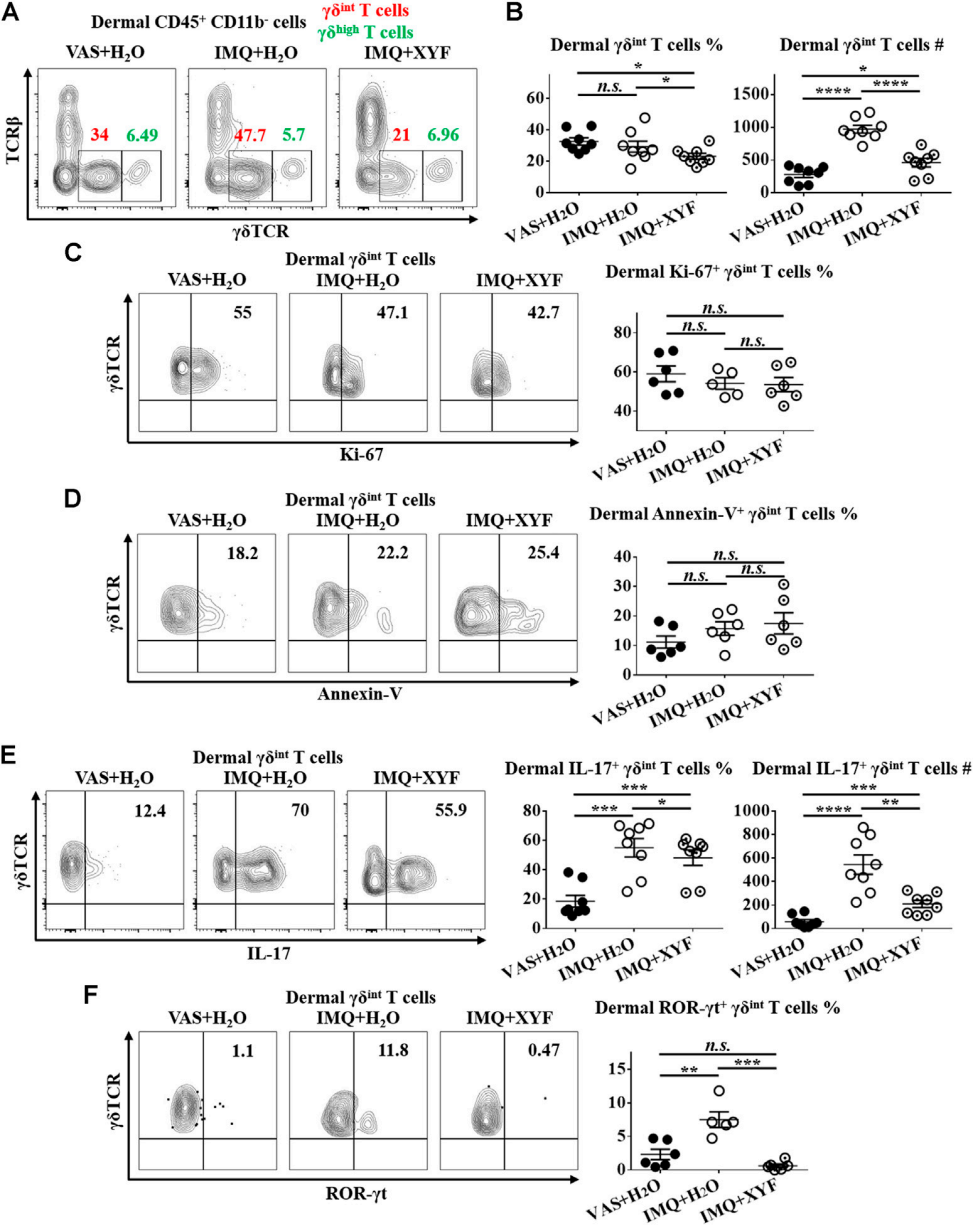
Xiao-Yin-Fang suppressed dermal γδT17 cell polarization. Mice were treated as in Figure 4, and the dermal cells of mouse ears were harvested. **(A–C)** Freshly isolated dermal cells were *in vitro* cultured in the presence of PMA, ionomycin and Brefeldin A for 4 h. Cultured dermal cells were stained with anti-CD45, TCRβ, γδTCR, and CD11b antibodies (*n* = 24, four independent experiments) **(A)** Representative scatter plots of dermal γδ^int^ T cells and γδ^high^ T cells. **(B)** The ratios and numbers of dermal γδ^int^ T cells. **(C)** Uncultured dermal cells were stained with anti-CD45, TCRβ, γδTCR, CD11b and Ki-67 antibodies. Representative scatter plots and the ratios of dermal Ki-67^+^ γδ^int^ T cells (*n* = 17, three independent experiments). **(D)** Uncultured dermal cells were stained with anti-CD45, TCRβ, γδTCR, CD11b and Annexin-V antibodies. Representative scatter plots and the ratios of dermal Annexin-V^+^ γδ^int^ T cells (*n* = 18, three independent experiments). **(F)** Cultured dermal cells were stained with anti-CD45, TCRβ, γδTCR, CD11b, and IL-17 antibodies. Representative scatter plots, the ratios and numbers of dermal IL-17^+^ γδ^int^ T cells (*n* = 24, three independent experiments). **(G)** Uncultured dermal cells were stained with anti-CD45, TCRβ, γδTCR, CD11b, and ROR-γt antibodies. Representative scatter plots and the ratios of dermal ROR-γt^+^ γδ^int^ T cells (*n* = 17, three independent experiments). The data were presented as mean ± s.e.m.

Previous studies have uncovered that dermal γδT cells were predominantly γδ^int^ T cells with a minor population being γδ^high^ T cells (Cai et al., 2011). And, dermal γδ^int^ T cells were the main source of IL-17 in psoriasis-like dermatitis, whereas γδ^high^ T cells barely produced IL-17 (Cai et al., 2011; Riol-Blanco et al., 2014). Hence, we focused on dermal γδ^int^ T cells in the following research. While dermal γδ^int^ T cells were expanded in model mice, XYF diminished the quantity of γδ^int^ T cells (Figures 5A,B). The expressions of Ki-67 and Annexin-V in γδ^int^ T cells were comparable between model and XYF group (Figures 5C,D), suggesting that XYF did not influence γδ^int^ T cell survival. Thus, the decrease in γδ^int^ T cells triggered by XYF might result from its impact on cell trafficking. Remarkably, while IL-17 secretion by γδ^int^ T cells tripled in model mice versus control mice, XYF significantly repressed γδ^int^ T cell production of IL-17 (Figure 5E). In accordance, the expression of ROR-γt, which was a house-keeping transcription factor of γδT17 cells, was enhanced in γδ^int^ T cells from model mice, whereas XYF considerably downregulated its expression (Figure 5F). In total, XYF might ameliorate psoriasis-like skin inflammation mainly through hampering dermal γδ^int^ T cell trafficking and suppressing their polarization into γδT17 cells.

### Xiao-Yin-Fang Alleviates The Relapse of Psoriasis-Like Dermatitis and Prohibits Dermal γδ^int^ T Cell Reactivation.

**FIGURE 6:**
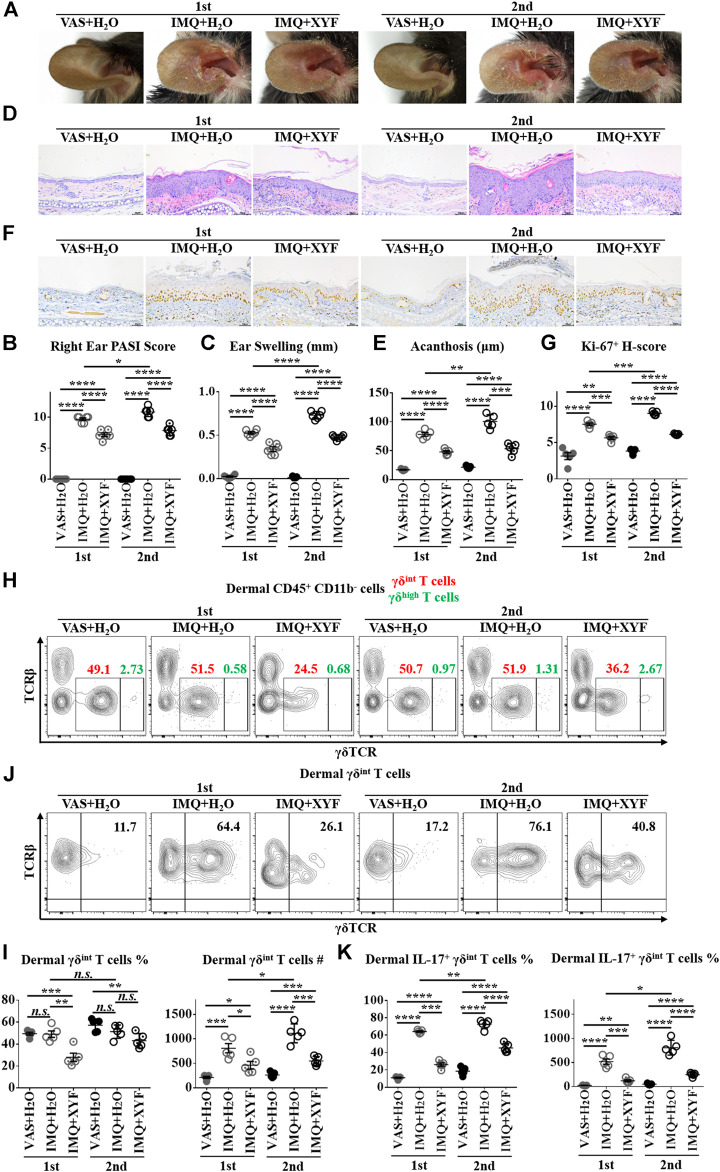
Xiao-Yin-Fang alleviated the relapse of psoriasis-like dermatitis and prohibited dermal γδ^int^ T cell reactivation. Mice were treated as in Supplementary Figure S7. **(A)** Representative pictures of mouse right ear lesions (1st: the initial occurrence of psoriasis-like dermatitis; 2nd: the recurrence of psoriasis-like dermatitis). **(B, C)** Evaluation of PASI score **(B)** and skin swelling **(C)** of mouse right ears (*n* = 36, two independent experiments). **(D)** H&E staining and calculated epidermal acanthosis **(E)** (x200; bar = 50 μm; *n* = 30, two independent experiments). **(F)** Ki-67 staining and assessment of its H-score of epidermal fields **(G)** (x200; bar = 50 μm; *n* = 30, two independent experiments). **(H-K)** Freshly isolated dermal cells were *in vitro* cultured in the presence of PMA, ionomycin and Brefeldin A for 4 h. Cultured dermal cells were stained with anti-CD45, TCRβ, γδTCR, CD11b, and IL-17 antibodies (*n* = 30, two independent experiments). **(H)** Representative scatter plots of dermal γδ^int^ T cells and γδ^high^ T cells. **(I)** The ratios and numbers of dermal γδ^int^ T cells. **(J)** Representative scatter plots of dermal IL-17^+^ γδ^int^ T cells. **(K)** The ratios and numbers of dermal IL-17^+^ γδ^int^ T cells. The data were presented as mean ± s.e.m.

As psoriasis has a notable propensity for recurrence, we exploited a relapsing model of psoriasis-like dermatitis to examine the therapeutic effects of XYF in psoriasis recurrence by applying IMQ cream on left ear and reapplying them on right ear one week later (Supplementary Figure S7) (Ramirez-Valle et al., 2015). As expected, the restimulation with IMQ exacerbated the severity of psoriasis-like dermatitis, which was successfully lessened by the treatment with XYF (Figure 6A). Evaluations of PASI score, ear swelling, epidermal acanthosis and Ki-67 H-score confirmed the therapeutic effects of XYF (Figures 6B–G). Consistently, the secondary application of IMQ further augmented γδ^int^ T cells and increased their IL-17 production, whereas XYF decreased γδ^int^ T cells and suppressed their secretion of IL-17 (Figures 6H–J). In sum, XYF might be beneficial to prevent the relapse of psoriasis through impeding γδ^int^ T cell reactivation and their polarization into γδT17 cells.

### Transcriptional Analysis of Therapeutic Effects of Xiao-Yin-Fang

**FIGURE 7:**
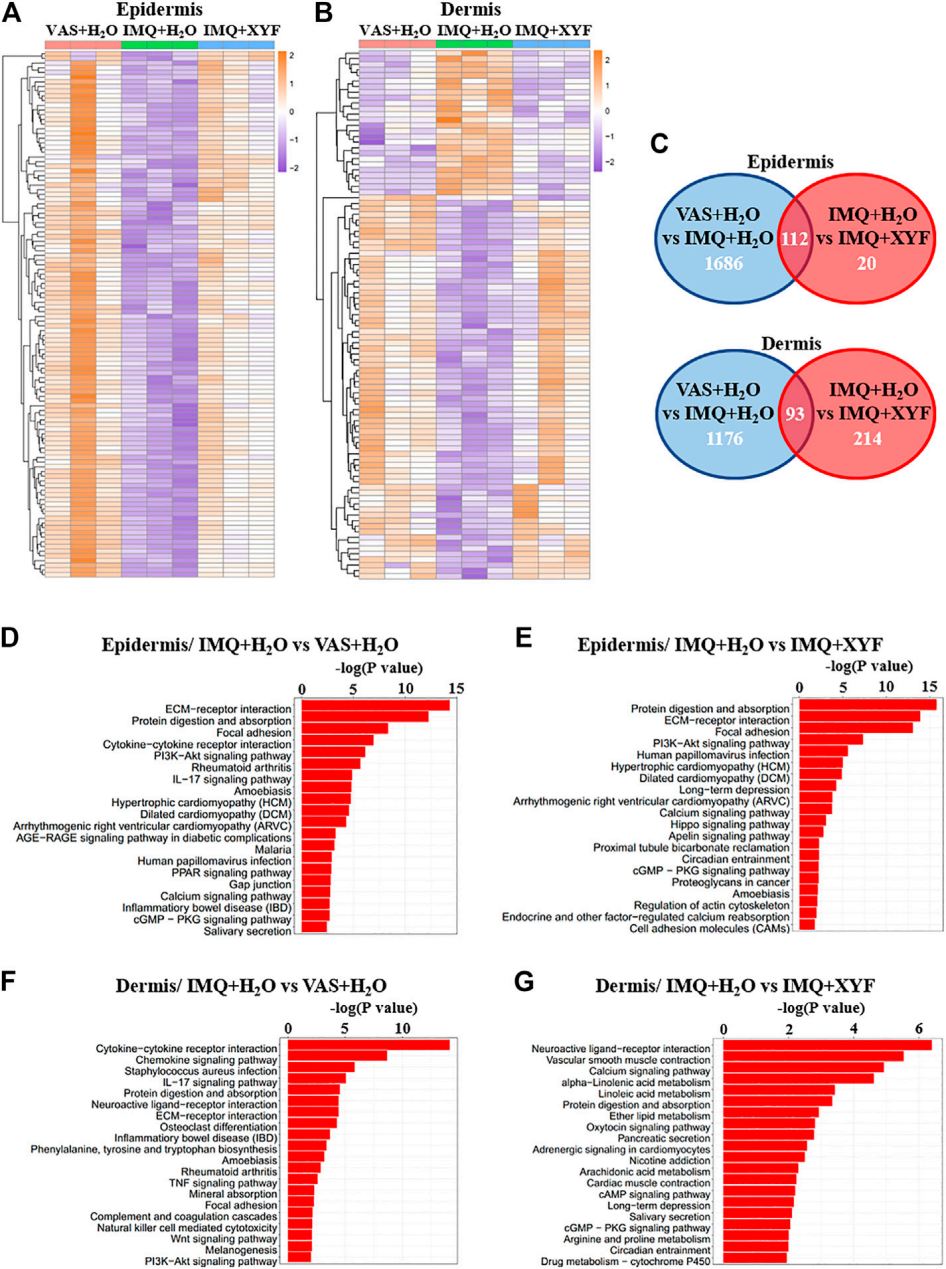
Transcriptional analysis of Xiao-Yin-Fang therapeutic effects. Epidermal and dermal sheets of mouse ears were freshly obtained from VAS + H_2_O, IMQ + H_2_O and IMQ + XYF group (*n* = 3 for each group), which underwent RNA sequencing analysis. **(A, B)** Heatmap clustering analysis of epidermal and dermal differentially expressed genes (DEG). **(C)** Venn diagrams showed the numbers of overlapping epidermal DEGs (upper panel) and dermal DEGs (lower panel) between VAS + H_2_O vs. IMQ + H_2_O and IMQ + H_2_O vs. IMQ + XYF. **(D-G)** KEGG pathway analysis of DEGs.

To further explore the potential mechanisms of XYF therapy, RNA sequencing analysis was performed (Supplementary Table S1). Heatmap analysis of differentially expressed genes (DEG) revealed distinct transcriptomes between different groups (Figures 7A,B). And, XYF partially reversed the pathological transcriptional alternations in psoriasis-like dermatitis (Figures 7A,B). Detailed comparison identified the overlapping genes of DEGs between control/model group and DEGs between model/XYF group (Figure 7C, Supplementary Table S2, S3). Kyoto Encyclopedia of Genes and Genomes (KEGG) pathway analysis uncovered that epidermis from model group was enriched in cytokine-cytokine receptor interaction, PI3K-Akt signaling pathway, IL-17 signaling pathway, and cGMP-PKG signaling pathway whereas the dermis from model group was enriched in cytokine-cytokine receptor interaction, chemokine signaling pathway, IL-17 signaling pathway, TNF signaling pathway, Wnt signaling pathway, and PI3K-Akt signaling pathway when compared with control group (Figures 7D,F). XYF therapy modulated PI3K-Akt signaling pathway, Calcium signaling, Hippo signaling pathway and cGMP-PKG signaling pathway in the epidermis, whereas it affected several metabolic pathways, Calcium signaling, cAMP signaling pathway and cGMP-PKG signaling pathway in the dermis (Figures 7E,G). Further Gene Set Enrichment Analysis (GSEA) uncovered XYF downregulated focal adhesion and ECM receptor interaction in the epidermis, whereas disturbed arachidonic acid metabolism in the dermis (Supplementary Figure S8). Altogether, these results suggested that XYF might regulate multiple inflammatory signaling pathways and metabolic processes, which impact on γδT17 cell biology awaits future study.

## Discussion

As one major population of skin-located innate immunocytes, dermal γδT cells might fundamentally contribute to the immunopathogenesis of psoriasis. Enrichment of γδT17 cells was observed in the dermis of psoriatic skin lesions (Cai et al., 2011). A subclass of Vγ9Vδ2^+^ T cells, which expressed IL-17 and tumor necrosis factor α (TNF-α), was distributed in normal skin from healthy individuals, non-lesional and lesional skin of psoriatic patients in increasing order (Laggner et al., 2011). In addition, the ratio of Vγ9Vδ2^+^ γδT cells were negatively correlated with the disease severity, indicating that Vγ9Vδ2^+^ γδT cell population might be recruited from the blood circulation to the cutaneous tissue when disease flares (Laggner et al., 2011). In line with the findings in psoriasis patients, γδT17 cells also played a dominant role in psoriasis-like dermatitis mouse model. The IL-17-secreting dermal cells were substantially decreased in TCRδ^−/−^ mice accompanied with lessened psoriasiform symptoms, whereas TCRα^−/−^ mice normally develop dermatitis (Cai et al., 2011). Previous studies have uncovered that IL-17 was mostly limited to dermal γδ^int^ T cells (Cai et al., 2011; Riol-Blanco et al., 2014). The IL-17-producing γδ^int^ T cells in the mouse dermis was primarily composed of Vγ4^+^ and Vγ6^+^ subsets (Cai et al., 2014; O'Brien and Born, 2015; Akitsu and Iwakura, 2018). Amongst, Vγ4^+^ T cells produced more IL-17 than Vγ6^+^ T cells, indicating that Vγ4^+^ T cells might play a more essential role in psoriasis-like dermatitis than Vγ6^+^ T cells (Cai et al., 2014). Moreover, pathogenic Vγ4^+^ T cells expanded and persisted within the dermis for a long time after initial exposure to IMQ, and these experienced γδT cells demonstrated heightened effector functions and aggravated secondary inflammation (Hartwig et al., 2015). Furthermore, Vγ4^+^ T cells traveled to noninflamed skin and peripheral LNs whey they exacerbated psoriasiform dermatitis at distant sites (Ramirez-Valle et al., 2015). The quasi-innate memory capacity of γδT cells might provide a novel mechanistic insight into psoriasis relapse. In summary, although multiple cellular sources of IL-17 have been identified in psoriasis, γδT cells might represent a potent contributor in the pathogenesis of psoriasis (Keijsers et al., 2014; Durham et al., 2015; Blauvelt and Chiricozzi, 2018). Therefore, the investigation of γδT17-targeted medication is of great importance in the management of psoriasis.

Xiao-Yin-Fang (XYF) is comprised of five Chinese herb medicines, involving *Isatis tinctoria L.* (Banlangen), *Scutellaria baicalensis Georgi* (Huangqin), *Salvia miltiorrhiza Bunge* (Danshen), *Sophora flavescens Aiton.* (Kushen), and *Rheum officinale Baill* (Dahuang). As bacterial infection has been reported to trigger and aggravate psoriasis, Banlangen contained multiple organic acids with potent antimicrobial activities, which included syringic acid, 2-amino-benzoic acid, salicylic acid and benzoic acid (Kong et al., 2008a; Kong et al., 2008b). Besides, N, N′-dicyclohexyl-N-arachidonic acylurea, a highly unsaturated fatty acid from Balangen, could inhibit TCR-mediated PI3K-Akt signaling pathway, resulting in arrested cell cycle transition from G1 to S phase and lymphocyte hypoproliferation (An et al., 2020). Erucic acid, an active component of Banlangen, markedly reduced CD8^+^ cytotoxic T lymphocyte recruitment (Liang et al., 2020). It is possible that the chemical constituents of Banlangen might affect the immune function of γδT cells.

Former research on Huangqin centered on its major bioactive flavonoid baicalin, which is also the highest-level chemical compound in XYF. Previous studies suggest that baicalin has the advantage of multi-target actions in treating psoriasis (Kim et al., 2013; Liu et al., 2015; Wu et al., 2015b; Bae et al., 2016; Huang et al., 2016; Hung et al., 2018). Stimulation with baicalein *in vitro* hindered HaCaT cell growth and augmented their expressions of keratin 1 and 10 via the inhibition of ERK phosphorylation (Huang et al., 2016). Consistently, the topical application of baicalin cream dose-dependently promoted the orthokeratosis of granular layers (Wu et al., 2015b). Moreover, baicalin suppressed the expressions of MHC class I/II and costimulatory molecules as well as inhibited IL-12 production from lipopolysaccharide-activated DCs, which repressed Th1/Th2/Th17 but promoted Treg cell differentiation (Kim et al., 2013; Liu et al., 2015; Bae et al., 2016). It has been reported that baicalin cream lessened IMQ-induced psoriasis-like dermatitis, which was accompanied with less infiltration of γδT cells into the skin lesions (Hung et al., 2018). Whether baicalin directly regulate the immune activity of γδT17 cells remained unexplored.

Danshen exhibited anti-inflammatory and anti-proliferative functions in psoriasis studies (Zhang et al., 2014; May et al., 2015). In silico screening revealed that Danshen contained compounds modulating apoptosis regulator Bcl-2, Bcl-2-Associated X, Caspase-3 along with TNF-α and Prostaglandin G/H synthase 2 (May et al., 2015). Salvianolic acid B reduced psoriatic changes by inhibiting psoriatic inflammatory and keratin markers by abolishing PI3K/Akt signaling pathway (Wang et al., 2020). Tanshinone IIA hindered keratinocyte growth via cell cycle arrest and apoptosis (Li et al., 2012). Cryptotanshinone (CTS), an active component of Danshen with antibacterial and antineoplastic effects, considerably relieved IMQ-induced epidermal hyperplasia through inhibiting STAT3-induced keratinocyte growth (Tang et al., 2018). Danshensu, the most abundant water-soluble component of Danshen, prevented abnormal keratinocyte proliferation in psoriasis by modulating YAP expression (Jia et al., 2020). The impact of Danshen on the immunofunctions of T lymphocytes require further investigation.

Matrine and oxymatrine, two main active phytocomponents of Kushen, have therapeutic potentials for psoriasis. Matrine lessened IMQ-induced psoriasiform cutaneous lesions by decreasing keratinocyte proliferation and MyD88 expression on the surface of DCs derived from bone marrow (Li et al., 2018). Matrine synergized with acitretin to induce cell cycle arrest and autophagy in keratinocytes by regulating PI3K/Akt/mTOR pathway (Jiang et al., 2019). Oxymatrine ameliorated skin inflammation in the patients with psoriasis and psoriasis-like mouse model via inhibiting keratinocyte proliferation probably through MAPK signaling pathway (Chen et al., 2017; Zhou et al., 2017; Shi et al., 2019; Xiang et al., 2020). Whether Kushen or its active components influence T cell biology remains unclear.

Emodin, which is a natural anthraquinone derivative of Dahuang, possesses broad-spectrum pharmacological properties, including antineoplastic, hepatoprotective, anti-inflammatory, antioxidant, and antimicrobial functions. The topical application of a natural compound mixture (PSM) of herbs, containing emodin, genipin, chlorogenic acid, cimigenoside, and ginsenoside Rb1, alleviated IMQ-induced psoriasis-like dermatitis and reduced the proliferation rate of IL-22-stimulated keratinocytes (Nguyen et al., 2020b). The therapeutic effects of Dahuang and its chemical compounds in psoriasis requires further study.

The RNA sequencing analysis revealed that XYF might regulate multiple inflammatory signaling pathways and metabolic processes. Amongst, PI3K-Akt, Calcium, and cAMP signaling pathways have been reported to influence γδT cell biology (Takano et al., 1998; Chen et al., 2005; Chen et al., 2020). Selective inhibition of PI3Kδ by Seletalisib hampered the production of IL-17 from peripheral blood γδT cells (Chen et al., 2020). Vitamin D, which profoundly regulates calcium metabolism, hindered the proinflammatory activity of γδT cells in a dose-dependent fashion (Chen et al., 2005). Prostaglandin E2 inhibited γδT cell cytotoxicity triggered by TCR receptors Vγ9Vδ2, NKG2D, and CD16 through a cAMP-mediated PKA type I-dependent signaling (Takano et al., 1998). Whether XYF suppress γδT17 cell polarization through these signaling pathways awaits future exploration.

In conclusion, XYF alleviated psoriasis-like skin inflammation mainly through suppressing dermal and draining lymph-node γδT17 cell polarization. Moreover, XYF therapy ameliorated the relapse of psoriasis-like dermatitis and prohibited dermal γδT cell reactivation. Transcriptional analysis suggested that XYF might regulate various inflammatory signaling and metabolic processes. Our results clarified the therapeutic efficacy and inner mechanisms of XYF therapy in psoriasis, which might promote its clinical application in psoriasis patients and facilitate the development of novel anti-psoriasis drugs based on the bioactive components of XYF.

## Data Availability

The datasets presented in this study can be found in online repositories. The names of the repository/repositories and accession numbers can be found below: https://www.ncbi.nlm.nih.gov/geo/, GSE161084 https://www.ncbi.nlm.nih.gov/geo/, GSE161350.

## Abbreviations

CAM, complementary and alternative medicine; DC, dendritic cells; ddH_2_O, double-distilled H_2_O; DEG, differentially expressed genes; FC, fold change; GSEA, gene set enrichment analysis; GTW, multi-glycoside of Tripterygium wilfordii Hook. f.; H&E, hematoxylin-eosin; IL, interleukin; γδT17, IL-17-producing γδT cells; IMQ, imiquimod; IS, intensity score; KEGG, kyoto encyclopedia of genes and genomes; LN, lymph nodes; PASI, psoriasis area and severity index; PMA, phorbol-12-myristate-13-acetate; PS, proportion score; SEM, standard error of mean; SPF, specific pathogen-free; Tc17, T cytotoxic 17 cells; Th17, T helper 17 cells; TCM, traditional chinese medicine; UPLC-Q/TOF-MS, Ultra-high performance liquid chromatography with quadrupole time-of-flight mass spectrometry, VAS, vaseline; XYF, Xiao-Yin-Fang.

## References

[B1] Akitsu A. Iwakura Y. (2018). Interleukin-17-producing γδ T (γδ 17) cells in inflammatory diseases. Immunology 155, 418–426. 10.1111/imm.12993 30133701 PMC6231014

[B2] An K. Qin Q. Yu S. Xue M. Wang Z. Lin Q. (2020). Combination of N , N ′‐dicyclohexyl‐ N ‐arachidonic acylurea and tacrolimus prolongs cardiac allograft survival in mice. Immunol. Cel Biol. 98, 382–396. 10.1111/imcb.12327 32162358

[B3] Bae M-J. Shin H. S. See H-J. Jung S. Y. Kwon D-A. Shon D-H. (2016). Baicalein induces CD4+Foxp3+ T cells and enhances intestinal barrier function in a mouse model of food allergy. Sci. Rep. 6, 32225. 10.1038/srep32225 27561877 PMC4999817

[B4] Blauvelt A. Chiricozzi A. (2018). The immunologic role of IL-17 in psoriasis and psoriatic arthritis pathogenesis. Clinic Rev. Allerg Immunol. 55, 379–390. 10.1007/s12016-018-8702-3 PMC6244934 30109481

[B5] Cai Y. Shen X. Ding C. Qi C. Li K. Li X. (2011). Pivotal role of dermal IL-17-producing γδ T cells in skin inflammation. Immunity 35, 596–610. 10.1016/j.immuni.2011.08.001 21982596 PMC3205267

[B6] Cai Y. Fleming C. Yan J. (2013). Dermal γδ T cells - a new player in the pathogenesis of psoriasis. Int. Immunopharmacology 16, 388–391. 10.1016/j.intimp.2013.02.018 23499509

[B7] Cai Y. Xue F. Fleming C. Yang J. Ding C. Ma Y. (2014). Differential developmental requirement and peripheral regulation for dermal Vγ4 and Vγ6T17 cells in health and inflammation. Nat. Commun. 5, 3986. 10.1038/ncomms4986 24909159 PMC4068267

[B8] Casciano F. Pigatto P. D. Secchiero P. Gambari R. Reali E. (2018). T cell hierarchy in the pathogenesis of psoriasis and associated cardiovascular comorbidities. Front. Immunol. 9, 1390. 10.3389/fimmu.2018.01390 29971067 PMC6018171

[B9] Chen L. Cencioni M. T. Angelini D. F. Borsellino G. Battistini L. Brosnan C. F. (2005). Transcriptional profiling of γδ T cells identifies a role for vitamin D in the immunoregulation of the Vγ9Vδ2 response to phosphate-containing ligands. J. Immunol. 174, 6144–6152. 10.4049/jimmunol.174.10.6144 15879110

[B10] Chen Q. Zhou H. Yang Y. Chi M. Xie N. Zhang H. (2017). Investigating the potential of Oxymatrine as a psoriasis therapy. Chemico-Biological Interactions 271, 59–66. 10.1016/j.cbi.2017.04.020 28450041

[B11] Chen S. Paveley R. Kraal L. Sritharan L. Stevens E. Dedi N. (2020). Selective targeting of PI3Kδ suppresses human IL-17-producing T cells and innate-like lymphocytes and may be therapeutic for IL-17-mediated diseases. J. Autoimmun. 111, 102435. 10.1016/j.jaut.2020.102435 32360069

[B12] Damevska K. Neloska L. Nikolovska S. Gocev G. Duma S. (2014). Complementary and alternative medicine use among patients with psoriasis. Dermatol. Ther. 27, 281–283. 10.1111/dth.12139 24964349

[B13] Diani M. Altomare G. Reali E. (2015). T cell responses in psoriasis and psoriatic arthritis. Autoimmun. Rev. 14, 286–292. 10.1016/j.autrev.2014.11.012 25445403

[B14] Durham L. E. Kirkham B. W. Taams L. S. (2015). Contribution of the IL-17 pathway to psoriasis and psoriatic arthritis. Curr. Rheumatol. Rep. 17, 55. 10.1007/s11926-015-0529-9 26209291

[B15] Farahnik B. Sharma D. Alban J. Sivamani R. (2017). Oral (systemic) botanical agents for the treatment of psoriasis: a review. J. Altern. Complement. Med. 23, 418–425. 10.1089/acm.2016.0324 28157393

[B16] Han R. Rostami-Yazdi M. Gerdes S. Mrowietz U. (2012). Triptolide in the treatment of psoriasis and other immune-mediated inflammatory diseases. Br. J. Clin. Pharmacol. 74, 424–436. 10.1111/j.1365-2125.2012.04221.x 22348323 PMC3477344

[B17] Hartwig T. Pantelyushin S. Croxford A. L. Kulig P. Becher B. (2015). Dermal IL-17-producing γδ T cells establish long-lived memory in the skin. Eur. J. Immunol. 45, 3022–3033. 10.1002/eji.201545883 26332438

[B18] Huang K-F. Ma K-H. Liu P-S. Chen B-W. Chueh S-H. (2016). Baicalein increases keratin 1 and 10 expression in HaCaT keratinocytes via TRPV4 receptor activation. Exp. Dermatol. 25, 623–629. 10.1111/exd.13024 27060689

[B19] Hung C-H. Wang C-N. Cheng H-H. Liao J-W. Chen Y-T. Chao Y-W. (2018). Baicalin ameliorates imiquimod-induced psoriasis-like inflammation in mice. Planta. Med. 84, 1110–1117. 10.1055/a-0622-8242 29763944

[B20] Jia J. Mo X. Liu J. Yan F. Wang N. Lin Y. (2020). Mechanism of danshensu-induced inhibition of abnormal epidermal proliferation in psoriasis. Eur. J. Pharmacol. 868, 172881. 10.1016/j.ejphar.2019.172881 31866405

[B21] Jiang W-W. Wang Y-M. Wang X-Y. Zhang Q. Zhu S-M. Zhang C-L. (2019). Role and mechanism of matrine alone and combined with acitretin for HaCaT cells and psoriasis-like murine models. Chin. Med. J. (Engl.) 132, 2079–2088. 10.1097/CM9.0000000000000412 31460901 PMC6793800

[B22] Keijsers R. R. M. C. Joosten I. Van Erp P. E. J. Koenen H. J. P. M. Van De Kerkhof P. C. M. (2014). Cellular sources of IL-17 in psoriasis: a paradigm shift? Exp. Dermatol. 23, 799–803. 10.1111/exd.12487 25039885

[B23] Kim J. Krueger J. G. (2017). Highly effective new treatments for psoriasis target the IL-23/type 17 T cell autoimmune *Axis* . Annu. Rev. Med. 68, 255–269. 10.1146/annurev-med-042915-103905 27686018

[B24] Kim M. E. Kim H. K. Park H.-Y. Kim D. H. Chung H. Y. Lee J. S. (2013). Baicalin from Scutellaria baicalensis impairs Th1 polarization through inhibition of dendritic cell maturation. J. Pharmacol. Sci. 121, 148–156. 10.1254/jphs.12200fp 23419270

[B25] Kong W. Zhao Y. Shan L. Xiao X. Guo W. (2008a). Microcalorimetric studies of the action on four organic acids in Radix isatidis on the growth of microorganisms. Chin. J. Biotechnol. 24, 646–650. 10.1016/s1872-2075(08)60033-3 18616177

[B26] Kong W-J. Zhao Y-L. Shan L-M. Xiao X-H. Guo W-Y. (2008b). Investigation on the spectrum-effect relationships of EtOAc extract from Radix Isatidis based on HPLC fingerprints and microcalorimetry. J. Chromatogr. B. 871, 109–114. 10.1016/j.jchromb.2008.06.053 18639503

[B27] Laggner U. Di Meglio P. Perera G. K. Hundhausen C. Lacy K. E. Ali N. (2011). Identification of a novel proinflammatory human skin-homing Vγ9Vδ2 T cell subset with a potential role in psoriasis. J. Immun. 187, 2783–2793. 10.4049/jimmunol.1100804 21813772 PMC3187621

[B28] Lebwohl M. G. Bachelez H. Barker J. Girolomoni G. Kavanaugh A. Langley R. G. (2014). Patient perspectives in the management of psoriasis: results from the population-based multinational assessment of psoriasis and psoriatic arthritis survey. J. Am. Acad. Dermatol. 70, 871–881. e830. 10.1016/j.jaad.2013.12.018 24576585

[B29] Li F.-L. Xu R. Zeng Q.-C. Li X. Chen J. Wang Y.-F. (2012). Tanshinone IIA inhibits growth of keratinocytes through cell cycle arrest and apoptosis: underlying treatment mechanism of psoriasis. Evidence-Based Complement. Altern. Med. 2012, 1. 10.1155/2012/927658 PMC3236062 22203883

[B30] Li N. Zhao J. Di T. Meng Y. Wang M. Li X. (2018). Matrine alleviates imiquimod-induced psoriasiform dermatitis in BALB/c mice via dendritic cell regulation. Int. J. Clin. Exp. Pathol. 11, 5232–5240. 31949603 PMC6963024

[B31] Liang X. Huang Y. Pan X. Hao Y. Chen X. Jiang H. (2020). Erucic acid from Isatis indigotica Fort. suppresses influenza A virus replication and inflammation *in vitro* and *in vivo* through modulation of NF-κB and p38 MAPK pathway. J. Pharm. Anal. 10, 130–146. 10.1016/j.jpha.2019.09.005 32373385 PMC7192973

[B32] Liu T. Dai W. Li C. Liu F. Chen Y. Weng D. (2015). Baicalin alleviates silica-induced lung inflammation and fibrosis by inhibiting the Th17 response in C57bl/6 mice. J. Nat. Prod. 78, 3049–3057. 10.1021/acs.jnatprod.5b00868 26605988

[B33] Lv M. Deng J. Tang N. Zeng Y. Lu C. (2018). Efficacy and safety of Tripterygium wilfordii Hook F on psoriasis vulgaris: a systematic review and meta-analysis of randomized controlled trials. Evidence-Based Complement. Altern. Med. 2018, 1. 10.1155/2018/2623085 PMC5937555 29849698

[B34] Magin P. J. Adams J. Heading G. S. Pond D. C. Smith W. (2006). Complementary and alternative medicine therapies in acne, psoriasis, and atopic eczema: results of a qualitative study of patients' experiences and perceptions. J. Altern. Complement. Med. 12, 451–457. 10.1089/acm.2006.12.451 16813509

[B35] May B. H. Deng S. Zhang A. L. Lu C. Xue C. C. L. (2015). In silico database screening of potential targets and pathways of compounds contained in plants used for psoriasis vulgaris. Arch. Dermatol. Res. 307, 645–657. 10.1007/s00403-015-1577-8 26142738

[B36] Michalek I. M. Loring B. John S. M. (2017). A systematic review of worldwide epidemiology of psoriasis. J. Eur. Acad. Dermatol. Venereol. 31, 205–212. 10.1111/jdv.13854 27573025

[B37] Murphy E. C. Nussbaum D. Prussick R. Friedman A. J. (2019). Use of complementary and alternative medicine by patients with psoriasis. J. Am. Acad. Dermatol. 81, 280–283. 10.1016/j.jaad.2019.03.059 30935988

[B38] Nguyen T. Lestienne F. Cousy A. Mengeaud V. Castex‐Rizzi N. (2020a). Effective inhibition of Th17/Th22 pathway in 2D and 3D human models of psoriasis by Celastrol enriched plant cell culture extract. J. Eur. Acad. Dermatol. Venereol. 34 (Suppl. 6), 3–9. 10.1111/jdv.16475 32783265

[B39] Nguyen U. T. Nguyen L. T. H. Kim B-A. Choi M-J. Yang I-J. Shin H-M. (2020b). Natural compound mixture, containing emodin, genipin, chlorogenic acid, cimigenoside, and ginsenoside Rb1, ameliorates psoriasis-like skin lesions by suppressing inflammation and proliferation in keratinocytes. Evidence-Based Complement. Altern. Med. 2020, 1. 10.1155/2020/9416962 PMC7603578 33149756

[B40] O’brien R. L. Born W. K. (2015). Dermal γδ T cells--What have we learned? Cell Immunol. 296, 62–69. 10.1016/j.cellimm.2015.01.011 25649119 PMC4466165

[B41] Pilon D. Teeple A. Zhdanava M. Ladouceur M. Ching Cheung H. Muser E. (2019). The economic burden of psoriasis with high comorbidity among privately insured patients in the United States. J. Med. Econ. 22, 196–203. 10.1080/13696998.2018.1557201 30523738

[B42] Ramírez-Valle F. Gray E. E. Cyster J. G. (2015). Inflammation induces dermal Vγ4+ γδT17 memory-like cells that travel to distant skin and accelerate secondary IL-17-driven responses. Proc. Natl. Acad. Sci. USA. 112, 8046–8051. 10.1073/pnas.1508990112 26080440 PMC4491769

[B43] Rapalli V. K. Singhvi G. Dubey S. K. Gupta G. Chellappan D. K. Dua K. (2018). Emerging landscape in psoriasis management: from topical application to targeting biomolecules. Biomed. Pharmacother. 106, 707–713. 10.1016/j.biopha.2018.06.136 29990862

[B44] Riol-Blanco L. Ordovas-Montanes J. Perro M. Naval E. Thiriot A. Alvarez D. (2014). Nociceptive sensory neurons drive interleukin-23-mediated psoriasiform skin inflammation. Nature 510, 157–161. 10.1038/nature13199 24759321 PMC4127885

[B45] Ru Y. Li H. Zhang R. Luo Y. Song J. Kuai L. (2020). Role of keratinocytes and immune cells in the anti-inflammatory effects of Tripterygium wilfordii Hook. f. in a murine model of psoriasis. Phytomedicine 77, 153299. 10.1016/j.phymed.2020.153299 32823074

[B46] Schleicher S. M. (2016). Psoriasis. Clin. Podiatric Med. Surg. 33, 355–366. 10.1016/j.cpm.2016.02.004 27215156

[B47] Shi H. J. Zhou H. Ma A. L. Wang L. Gao Q. Zhang N. (2019). Oxymatrine therapy inhibited epidermal cell proliferation and apoptosis in severe plaque psoriasis. Br. J. Dermatol. 181, 1028–1037. 10.1111/bjd.17852 30822359 PMC6899633

[B48] Takano M. Nishimura H. Kimura Y. Washizu J. Mokuno Y. Nimura Y. (1998). Prostaglandin E2 protects against liver injury after Escherichia coli infection but hampers the resolution of the infection in mice. J. Immunol. 161, 3019–3025. 9743366

[B49] Tang L. He S. Wang X. Liu H. Zhu Y. Feng B. (2018). Cryptotanshinonereduces psoriatic epidermal hyperplasia via inhibiting the activation of STAT3. Exp. Dermatol. 27, 268–275. 10.1111/exd.13511 29427477

[B50] Wang W. Gao Z. Guo Z. Guo Q. Yang Q. Gu J. (2012a). Efficacy of Xiaoyin decoction combined with calcipotriol ointment in patients with vulgaris psoriasis of blood heat type and their effects on related cytokines. Chin. J. Dermatol. 45, 647–649.

[B51] Wang W. Wang F. Gao Z. Bu X. Gu J. (2012b). Clinical effects of xiaoyin tang in treating psoriasis of blood-heat type and its impact on T-bet/GATA3. Chin. J. Dermatovenerology Integrated Traditional West. Med. 11 (2), 74–77.

[B52] Wang S. Zhu L. Xu Y. Qin Z. Xu A. (2020). Salvianolic acid B ameliorates psoriatic changes in imiquimod-induced psoriasis on BALB/c mice by inhibiting inflammatory and keratin markers via altering phosphatidylinositol-3-kinase/protein kinase B signaling pathway. Korean J. Physiol. Pharmacol. 24, 213–221. 10.4196/kjpp.2020.24.3.213 PMC7193910 32392912

[B53] Wu C. Jin H-Z. Shu D. Li F. He C-X. Qiao J. (2015a). Efficacy and safety of Tripterygium wilfordii Hook F versus acitretin in moderate to severe psoriasis vulgaris. Chin. Med. J. (Engl.) 128, 443–449. 10.4103/0366-6999.151069 25673443 PMC4836244

[B54] Wu J. Li H. Li M. (2015b). Effects of baicalin cream in two mouse models: 2,4-dinitrofluorobenzene-induced contact hypersensitivity and mouse tail test for psoriasis. Int. J. Clin. Exp. Med. 8, 2128–2137. 25932143 PMC4402790

[B55] Xiang W. Suo T-C. Yu H. Li A-P. Zhang S-Q. Wang C-H. (2018). A new strategy for choosing “Q-markers” *via* network pharmacology, application to the quality control of a Chinese medical preparation. J. Food Drug Anal. 26, 858–868. 10.1016/j.jfda.2017.10.003 29567258 PMC9322248

[B56] Xiang X. Tu C. Li Q. Wang W. Huang X. Zhao Z. (2020). Oxymatrine ameliorates imiquimod-induced psoriasis pruritus and inflammation through inhibiting heat shock protein 90 and heat shock protein 60 expression in keratinocytes. Toxicol. Appl. Pharmacol. 405, 115209. 10.1016/j.taap.2020.115209 32835761

[B57] Zhang H. Xu X. G. Gu J. (2008). [Effect of xiaoyin recipe in treatment of psoriasis patients of blood-heat syndrome type and its impact on peripheral Th1/Th2 equilibrium]. Zhongguo Zhong Xi Yi Jie He Za Zhi 28, 683–685. 18928088

[B58] Zhang C. S. Yu J. J. Parker S. Zhang A. L. May B. Lu C. (2014). Oral Chinese herbal medicine combined with pharmacotherapy for psoriasis vulgaris: a systematic review. Int. J. Dermatol. 53, 1305–1318. 10.1111/ijd.12607 25208594

[B59] Zhao J. Di T. Wang Y. Liu X. Liang D. Zhang G. (2016). Multi-glycoside of Tripterygium wilfordii Hook. f. ameliorates imiquimod-induced skin lesions through a STAT3-dependent mechanism involving the inhibition of Th17-mediated inflammatory responses. Int. J. Mol. Med. 38, 747–757. 10.3892/ijmm.2016.2670 27431437 PMC4990293

[B60] Zhou H. Shi H-J. Yang J. Chen W-G. Xia L. Song H-B. (2017). Efficacy of oxymatrine for treatment and relapse suppression of severe plaque psoriasis: results from a single-blinded randomized controlled clinical trial. Br. J. Dermatol. 176, 1446–1455. 10.1111/bjd.15316 28112799

