# Supplementary material for: Xiao-Yin-Fang Therapy Alleviates Psoriasis-like Skin Inflammation Through Suppressing γδT17 Cell Polarization
Source: Front Pharmacol. 2021 Apr 16;12:629513. doi: 10.3389/fphar.2021.629513 (PMC8087247; doi:10.3389/fphar.2021.629513)
Supplement: Supplementary file 1 [file datasheet1.docx]

## Supplementary Figures

##
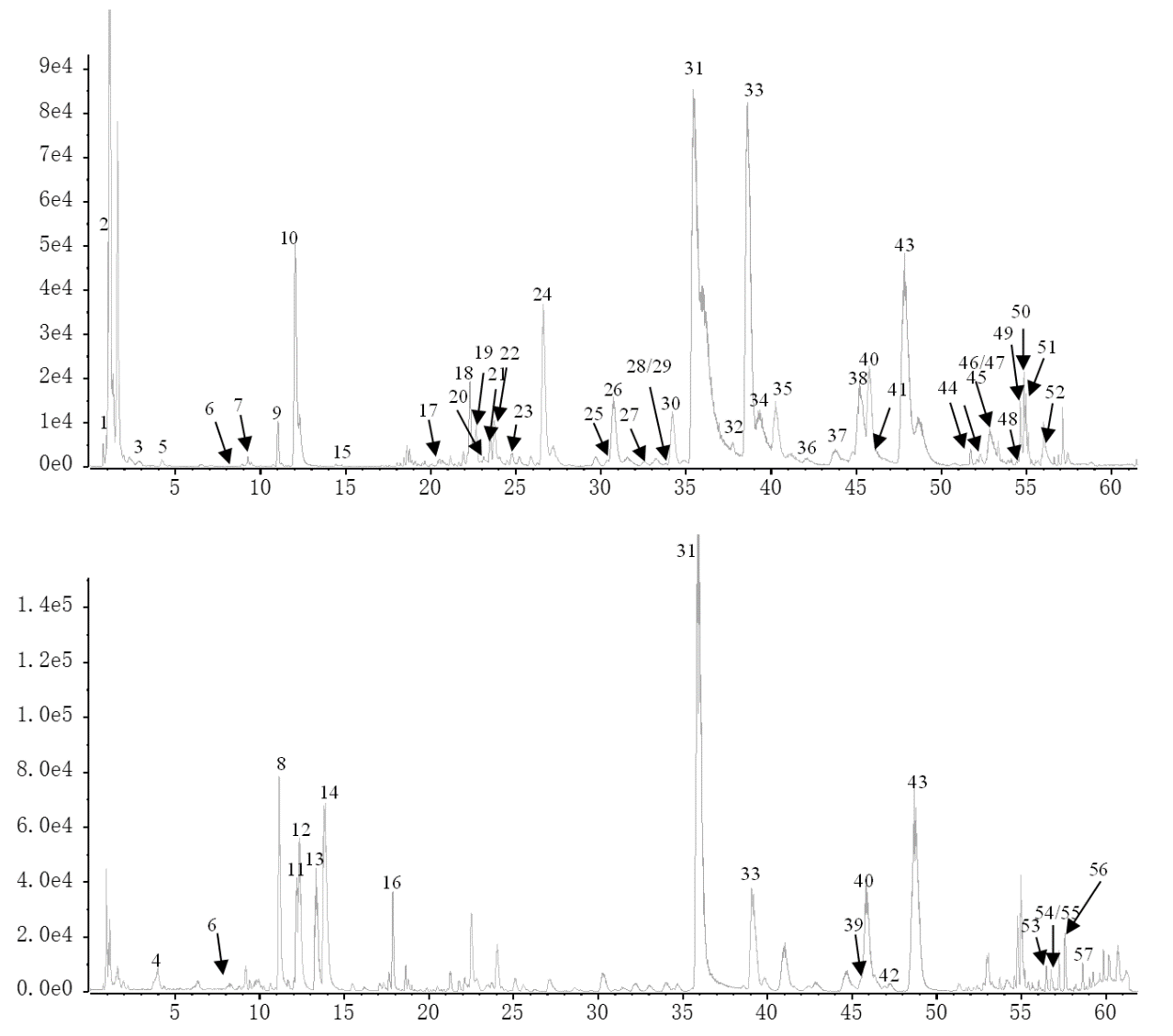


**Figure S1 UPLC-Q/TOF-MS profiling of Xiao-Yin-Fang**

Fifty-seven major chemical compositions in Xiao-Yin-Fang formula were detected via UPLC-Q/TOF-MS analysis, which did not contain conventional immunosuppressive agents.


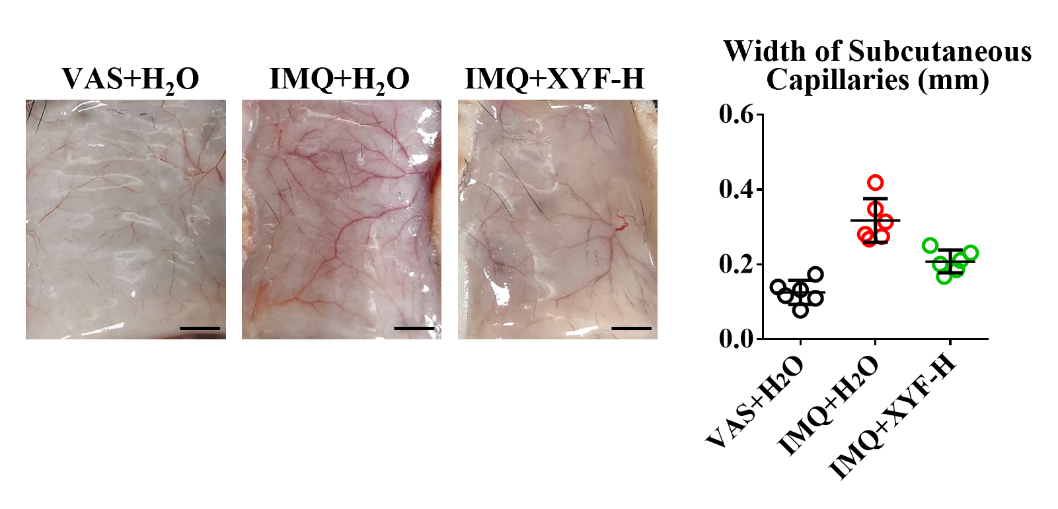


**Figure S2 Xiao-Yin-Fang lessened subcutaneous vessel dilation in psoriasis-like dermatitis**

Representative photographs of subcutaneous capillaries and their widths at the widest points measured by ImageJ software (bar=4mm; n=18, two independent experiments).


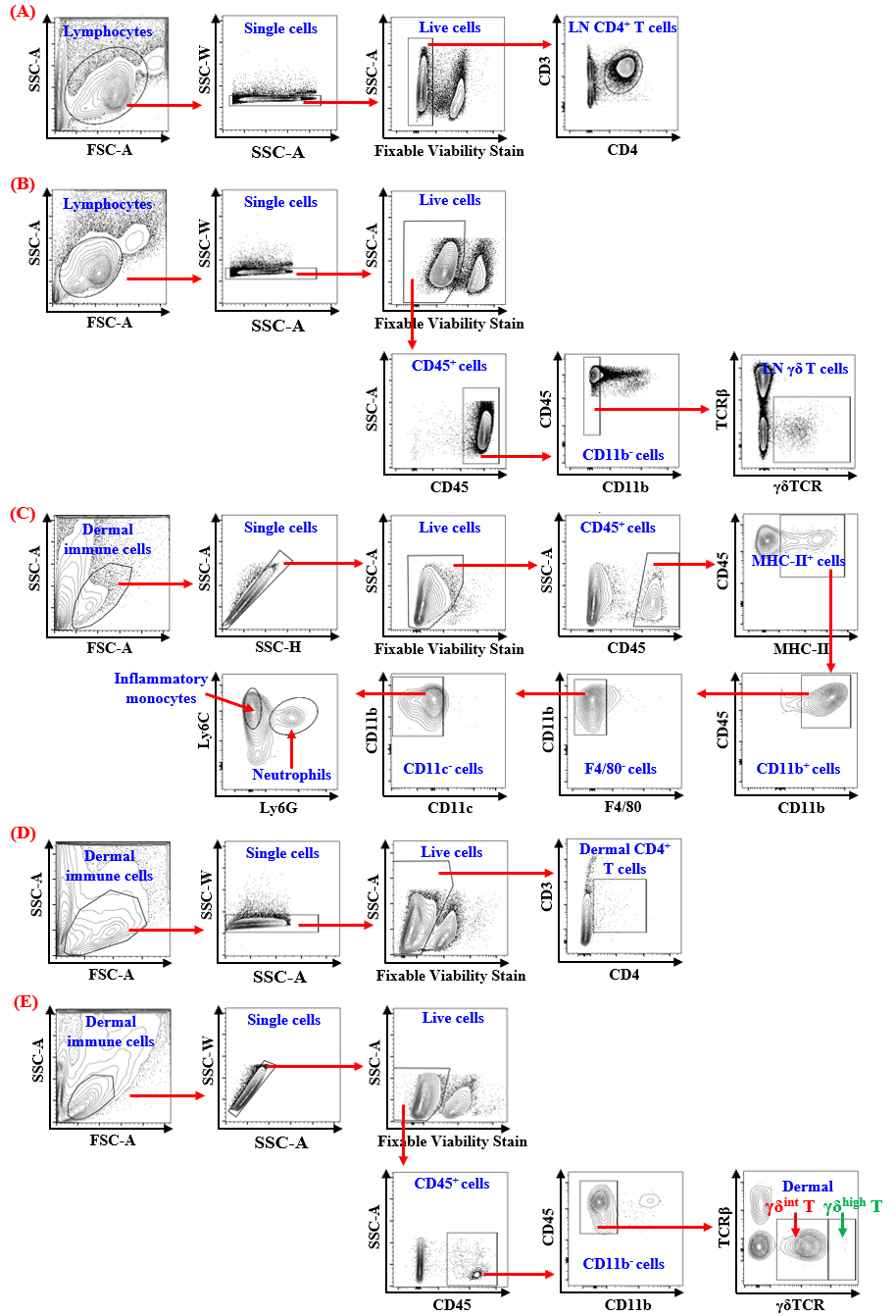


**Figure S3 Multistep flow cytometry strategy**

Gate strategy for LN CD4^+^ T cells **(A)**, LN γδT cells **(B)**, dermal neutrophils **(C)**, dermal inflammatory monocytes **(C)**, dermal CD4^+^ T cells **(D)**, and dermal γδT cells **(E)**.

##
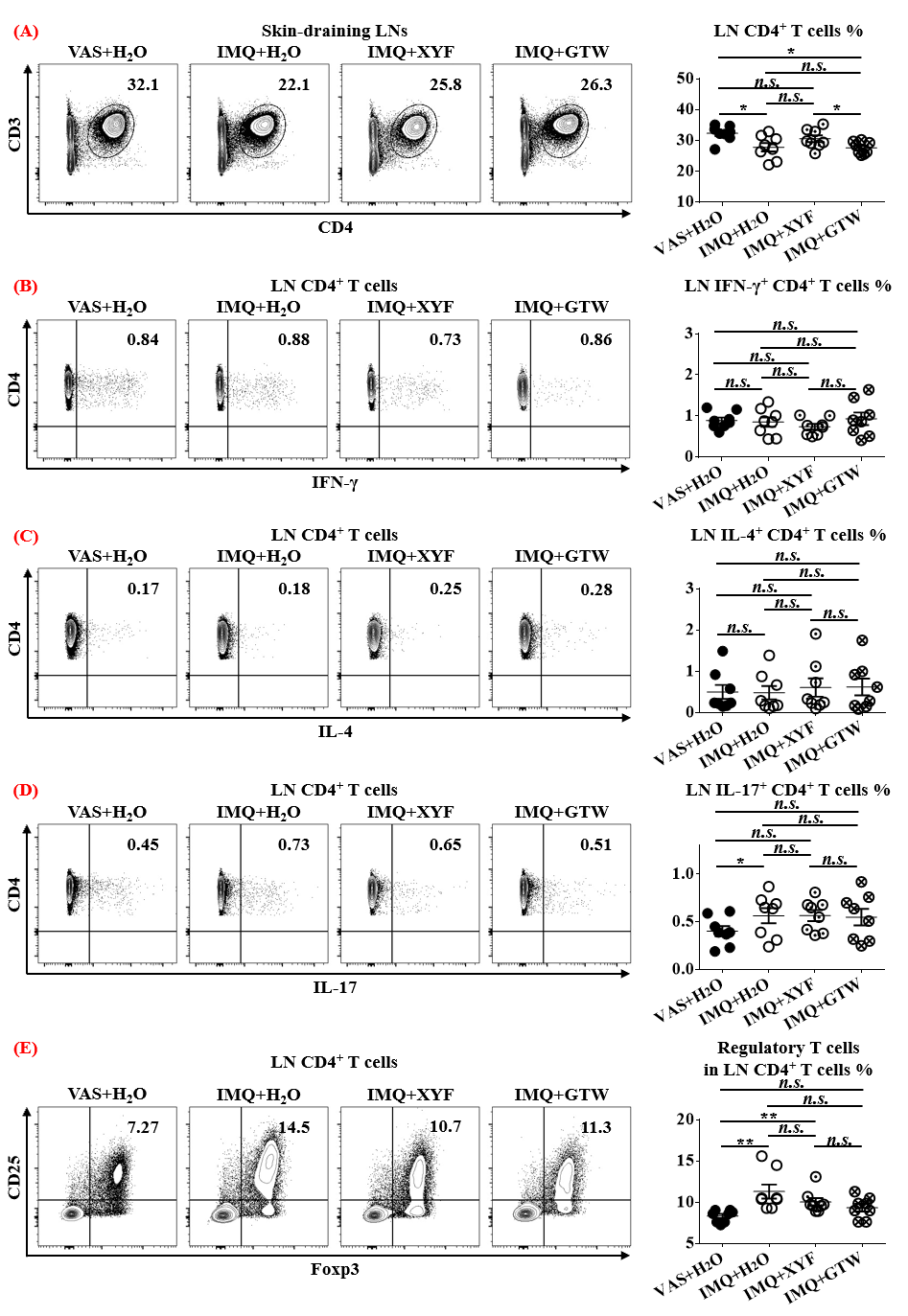


**Figure S4 Xiao-Yin-Fang did not influence T helper cells and regulatory T cells in the lymph nodes**

Mice were treated as in **Figure 2**. Skin-draining axillary, brachial and inguinal lymph nodes (LN) were harvested on day 10. **(A-D)** Freshly-isolated LN cells were *in vitro* cultured in the presence of PMA, ionomycin and Brefeldin A for 4 hours, and stained with anti-CD3, CD4, IFN-γ, IL-4 and IL-17 antibodies, which were analyzed by flow cytometry. Representative scatter plots and the ratio of LN CD4^+^ T cells **(A)**, IFN-γ^+^ CD4^+^ T cells **(B)**, IL-4^+^ CD4^+^ T cells **(C)** and IL-17^+^ CD4^+^ T cells **(D)**. **(E)** Freshly-isolated LN cells were stained with anti-CD3, CD4, CD25 and Foxp3 antibodies. Representative scatter plots and the percentage of LN regulatory T cells. n=32, three independent experiments. The data are presented as mean ± s.e.m.

**
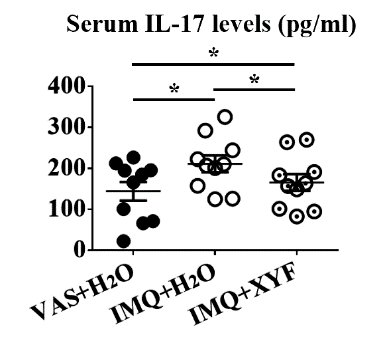
**

**Figure S5 Xiao-Yin-Fang reduced serum IL-17 level in model mice**

Mice were treated as in **Figure 2**. Mouse serums were collected on day 10. Quantitative evaluation of serum IL-17 level by enzyme-linked immunosorbent assay (ELISA). n=30, five independent experiments.

**
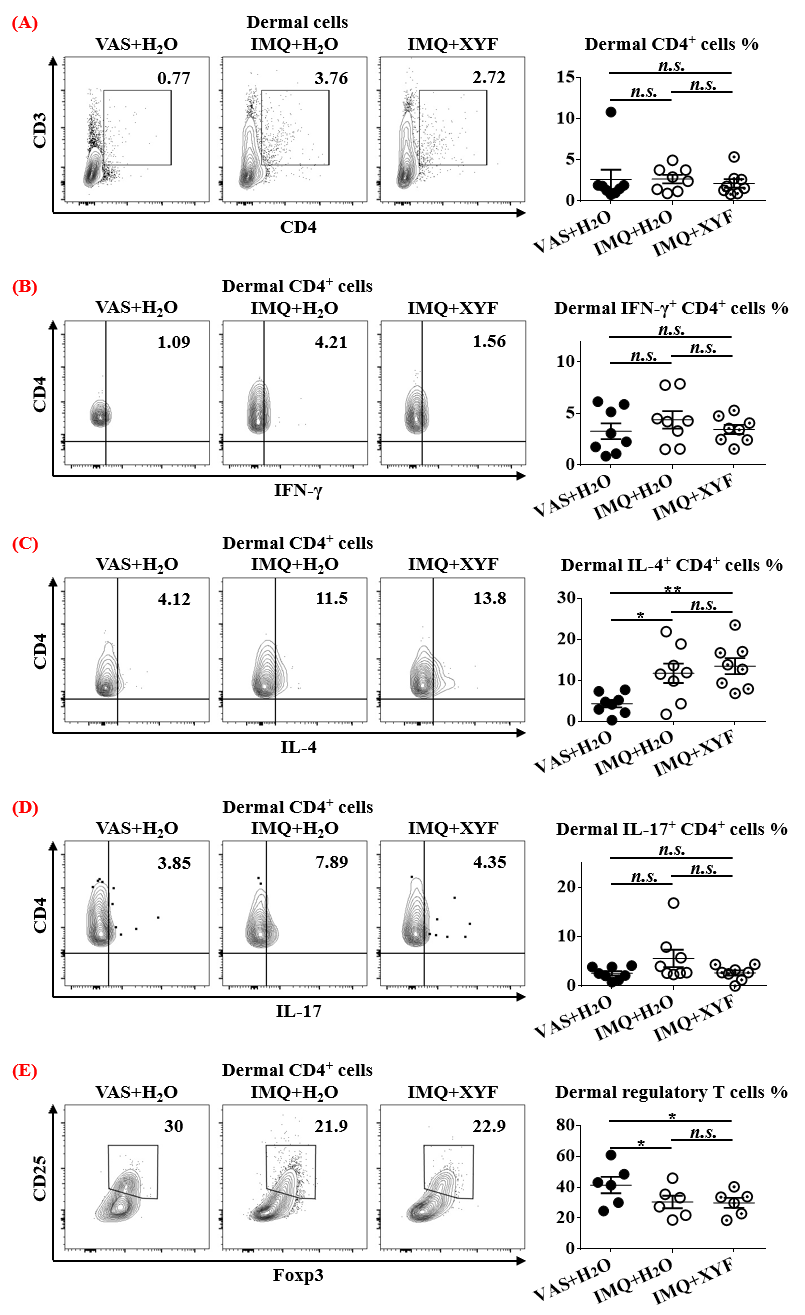
**

**Figure S6 Xiao-Yin-Fang did not affect dermal T helper cells and regulatory T cells**

Mice were treated as in **Figure 4**. **(A-D)** Freshly-isolated dermal cells were *in vitro* cultured in the presence of PMA, ionomycin and Brefeldin A for 4 hours, and stained with anti-CD3, CD4, IFN-γ, IL-4 and IL-17 antibodies, which were analyzed by flow cytometry. Representative scatter plots and the ratios of dermal CD4^+^ T cells (A), IFN-γ^+^ CD4^+^ T cells (B), IL-4^+^ CD4^+^ T cells (C) and IL-17^+^ CD4^+^ T cells (D) (n=24, four independent experiments). **(E)** Freshly-isolated dermal cells were stained with anti-CD3, CD4, CD25 and Foxp3 antibodies. Representative scatter plots and the percentages of dermal regulatory T cells (n=18, three independent experiments). The data are presented as mean ± s.e.m.

**
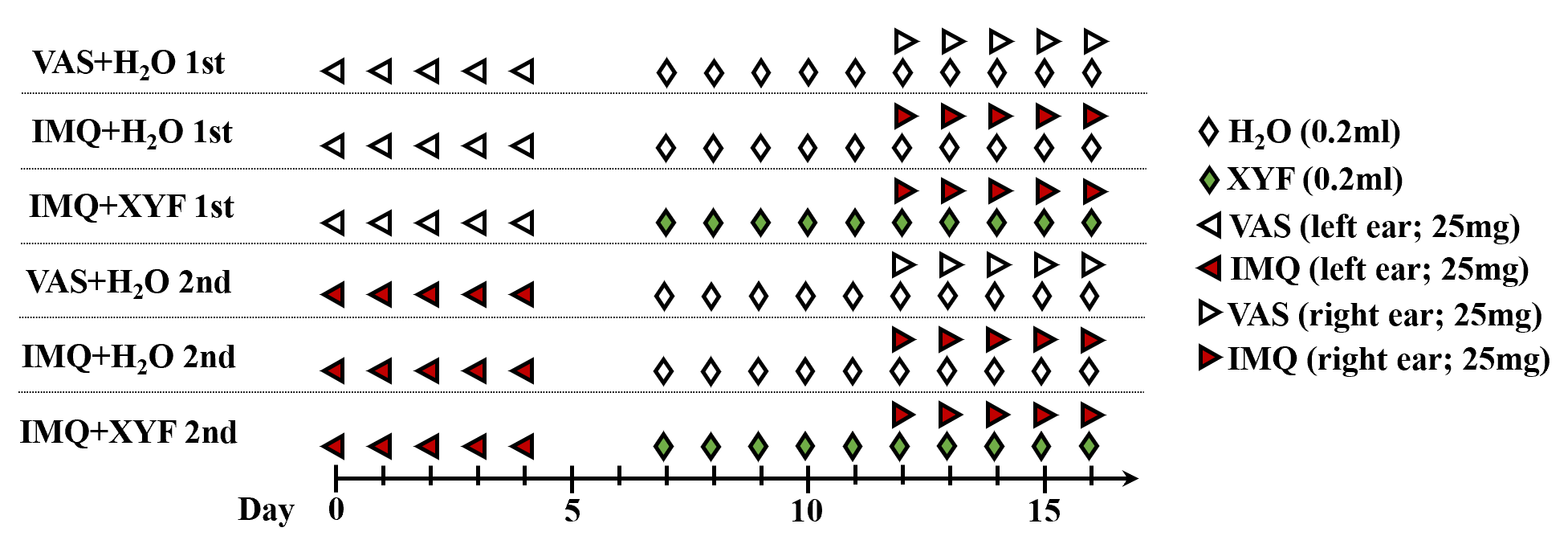
**

**Figure S7 Schematic representation of the experimental procedures of psoriasis relapse**

25mg of IMQ or VAS was applied on mouse left ear once daily from day 0 to day 4, XYF or H_2_O was administered by gavage twice daily from day 7 to day 16, and 25mg of IMQ or VAS was reapplied on mouse right ear once daily from day 12 to day 16.

**
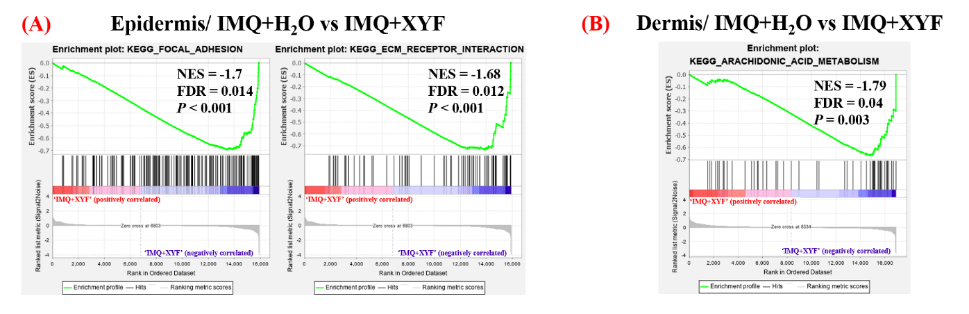
**

**Figure S8 Gene set enrichment analysis of Xiao-Yin-Fang therapeutic effects**

Epidermal and dermal sheets of mouse ears were freshly obtained from IMQ+H_2_O and IMQ+XYF group (n=3 for each group), which underwent RNA sequencing analysis. **(A)** Gene set enrichment analysis (GSEA) plots of epidermal DEGs from IMQ+H_2_O vs. IMQ+XYF. **(B)** GSEA plots of dermal DEGs from IMQ+ H_2_O vs. IMQ+XYF.

**Supplementary Tables**

**Table S1 The summary of raw RNA sequencing data**

It showed the summary of RNA sequencing data of 18 samples, including raw reads, clean reads, and percentage of clean reads as well as Q20% and Q30% (the percentage of bases with Phred values > 20 and > 30)

| **Sample** | **Raw Reads** | **Clean Reads** | **Clean Reads%** | **Q20%** | **Q30%** |
| --- | --- | --- | --- | --- | --- |
| VAS+H_2_O E1 | 60701562 | 60131004 | 99.06% | 98.55% | 95.55% |
| VAS+H_2_O E2 | 42160364 | 41690370 | 98.89% | 98.40% | 95.30% |
| VAS+H_2_O E3 | 46823034 | 46324902 | 98.94% | 98.45% | 95.35% |
| IMQ+H_2_O E4 | 40007914 | 39623684 | 99.04% | 98.45% | 95.40% |
| IMQ+H_2_O E5 | 51651208 | 51175616 | 99.08% | 98.45% | 95.40% |
| IMQ+H_2_O E6 | 58577918 | 57893658 | 98.83% | 98.35% | 95.20% |
| IMQ+XYF E7 | 83875478 | 83217278 | 99.22% | 98.65% | 95.85% |
| IMQ+XYF E8 | 43356836 | 42851552 | 98.83% | 98.40% | 95.15% |
| IMQ+XYF E9 | 45409532 | 45005498 | 99.11% | 98.55% | 95.55% |
| VAS+H_2_O D1 | 56746542 | 55864576 | 98.45% | 98.10% | 94.75% |
| VAS+H_2_O D2 | 49223016 | 48632398 | 98.80% | 98.35% | 95.15% |
| VAS+H_2_O D3 | 105123978 | 104335864 | 99.25% | 98.65% | 96% |
| IMQ+H_2_O D4 | 42108550 | 41492688 | 98.54% | 97.95% | 94.50% |
| IMQ+H_2_O D5 | 54698970 | 53883872 | 98.51% | 98.25% | 94.95% |
| IMQ+H_2_O D6 | 72507924 | 71686876 | 98.87% | 98.45% | 95.55% |
| IMQ+XYF D7 | 108048258 | 106963218 | 99% | 98.55% | 95.80% |
| IMQ+XYF D8 | 68720098 | 67956176 | 98.89% | 98.25% | 95.10% |
| IMQ+XYF D9 | 81647132 | 80877760 | 99.06% | 98.60% | 95.80% |

**Table S2 Epidermal shared DEGs between VAS+H_2_O vs. IMQ+H_2_O and IMQ+ H_2_O vs. IMQ+XYF and their associated dysregulated KEGG pathways**

| **KEGG Pathway Name** | **Symbol List** |
| --- | --- |
| ECM-receptor interaction | Fn1,Col6a3,Comp,Col6a1,Col6a2,Itga10,Col9a3,Itga1,Col2a1,Col1a2,Col9a1,Col9a2 |
| Focal adhesion | Pdgfrb,Fn1,Col6a3,Comp,Col6a1,Col6a2,Itga10,Col9a3,Itga1,Col2a1,Col1a2,Col9a1,Col9a2,Igf1 |
| PI3K-Akt signaling pathway | Pdgfrb,Fn1,Col6a3,Comp,Col6a1,Col6a2,Itga10,Col9a3,Itga1,Col2a1,Col1a2,Col9a1,Col9a2,Igf1 |
| Calcium signaling pathway | Pdgfrb,Plce1 |
| Hippo signaling pathway | Fzd9,Bmp5,Gdf5 |
| Circadian entrainment | Prkg1 |
| Proteoglycans in cancer | Fn1,Lum,Fzd9,Col1a2,Plce1,Igf1 |
| cGMP - PKG signaling pathway | Prkg1, |
| Regulation of actin cytoskeleton | Pdgfrb,Fn1,Itga10,Itga1 |
| Hematopoietic cell lineage | Itga1 |
| AGE-RAGE signaling pathway in diabetic complications | Fn1,Col1a2,Plce1 |
| Wnt signaling pathway | Fzd9,Wif1 |
| cAMP signaling pathway | Plce1 |

**Table S3 Dermal shared DEGs between VAS+ H_2_O vs. IMQ+ H_2_O and IMQ+H_2_O vs. IMQ+XYF and their associated dysregulated KEGG pathways**

| **Pathway Name** | **Symbol List** |
| --- | --- |
| Neuroactive ligand-receptor interaction | Gria1 |
| alpha-Linolenic acid metabolism | Pla2g5 |
| Linoleic acid metabolism | Pla2g5 |
| Ether lipid metabolism | Pla2g5 |
| Arachidonic acid metabolism | Alox12,Pla2g5 |
| cAMP signaling pathway | Gria1 |
| Arginine and proline metabolism | Pycr1 |
| Circadian entrainment | Gria1 |
| Inflammatory mediator regulation of TRP channels | Alox12 |
| Complement and coagulation cascades | C9 |
| Wnt signaling pathway | Wnt9b,Fzd9,Wif1,Wnt3a |
| Fc epsilon RI signaling pathway | Fcer1a |
| Long-term potentiation | Gria1 |
